# Ensemble learning and ground-truth validation of synaptic connectivity inferred from spike trains

**DOI:** 10.1371/journal.pcbi.1011964

**Published:** 2024-04-29

**Authors:** Christian Donner, Julian Bartram, Philipp Hornauer, Taehoon Kim, Damian Roqueiro, Andreas Hierlemann, Guillaume Obozinski, Manuel Schröter

**Affiliations:** 1 Swiss Data Science Center, ETH Zürich & EPFL, Zürich & Lausanne, Switzerland; 2 Department of Biosystems Science and Engineering, ETH Zürich, Basel, Switzerland; University of Connecticut, UNITED STATES

## Abstract

Probing the architecture of neuronal circuits and the principles that underlie their functional organization remains an important challenge of modern neurosciences. This holds true, in particular, for the inference of neuronal connectivity from large-scale extracellular recordings. Despite the popularity of this approach and a number of elaborate methods to reconstruct networks, the degree to which synaptic connections can be reconstructed from spike-train recordings alone remains controversial. Here, we provide a framework to probe and compare connectivity inference algorithms, using a combination of synthetic ground-truth and in vitro data sets, where the connectivity labels were obtained from simultaneous high-density microelectrode array (HD-MEA) and patch-clamp recordings. We find that reconstruction performance critically depends on the regularity of the recorded spontaneous activity, i.e., their dynamical regime, the type of connectivity, and the amount of available spike-train data. We therefore introduce an ensemble artificial neural network (eANN) to improve connectivity inference. We train the eANN on the validated outputs of six established inference algorithms and show how it improves network reconstruction accuracy and robustness. Overall, the eANN demonstrated strong performance across different dynamical regimes, worked well on smaller datasets, and improved the detection of synaptic connectivity, especially inhibitory connections. Results indicated that the eANN also improved the topological characterization of neuronal networks. The presented methodology contributes to advancing the performance of inference algorithms and facilitates our understanding of how neuronal activity relates to synaptic connectivity.

## Author summary

This study introduces an ensemble artificial neural network (eANN) to infer neuronal connectivity from spike times. We benchmark the eANN against existing connectivity inference algorithms and validate it using *in silico* simulations and *in vitro* data obtained from parallel high-density microelectrode array (HD-MEA) and patch-clamp recordings. Results demonstrate that the eANN outperforms all other algorithms across different dynamical regimes and provides a more accurate description of the underlying topological organization of the studied networks. Further examinations of the eANN’s output are conducted to identify which input features are most instrumental in achieving this enhanced performance. In sum, the eANN is a promising approach to improve connectivity inference from spike-train data.

This is a *PLOS Computational Biology* Methods paper.

## Introduction

Inferring the wiring diagram of complex neuronal circuits, their *connectomes*, has remained an important pillar in the quest to understand how individual neurons process information and how neuronal networks are organized [1]. Recent years have seen significant advances in connectome inference techniques enabling the study of fundamental principles of neuronal organization [2], and the intricate structure-function relationship of local synaptic connectivity [3, 4]. In particular, methods based on serial block-face electron microscopy (EM) [5], and virus-based circuit reconstruction [6], have paved the way to mapping out synaptic connections in unprecedented detail. These methods significantly deepened our understanding of how circuit architecture relates to neuronal communication across scales, i.e., at the level of local connectivity, as well as, across different brain regions. The interest in linking connectomics and functional readouts has also been fuelled by the ever-increasing capabilities of large-scale electrophysiological recording technology for studying neuronal physiology *in vivo* [7] and *in vitro* [8, 9].

A large body of studies, including different species and brain regions, has started to provide insight into the specific connectivity patterns that individual neurons form to communicate. Common organizational motifs of synaptic connectivity include, for example, feedforward excitation, feedforward inhibition, as well as, feedback inhibition, and lateral inhibition [10]. In addition to these circuit motifs, a range of complex topological properties have been described [2], among them, a greater-than-random community structure [11, 12], the occurrence of specific triple-motifs among locally connected projection neurons [13, 14], a small-world [15] and rich-club organization [16], and highly-connected hub neurons [17]. Studies also found that the synaptic strength of local circuitry typically follows a heavy-tailed log-normal distribution with few strong connections [18, 19]. Many of these synaptic wiring diagrams have been obtained through EM reconstruction in model organisms, such as *Caenorhabditis elegans* [20], *drosophila* [21], and *zebrafish* [22, 23], and more recently, through reconstruction of small tissue samples of mouse [4, 24], macaque and human cortex [25].

In addition to dense, EM-based reconstruction of neuronal circuits, which allows for the perhaps most comprehensive characterization of synaptic connectivity, important alternative circuit-mapping tools exist. The two most widely used techniques are viral retrograde and anterograde trans-synaptic labeling of neurons [6, 26], and whole-cell patch-clamp recordings [27]. Patch-clamp recordings are the gold standard to infer synaptic function and have been widely applied to characterize synaptic connections among pre- and postsynaptic neurons, including the strength of their excitatory and inhibitory postsynaptic potentials (EPSPs/IPSPs), the time course of the postsynaptic responses, and the reliability of synaptic transmission. To assess if two neurons are monosynaptically connected, typically, whole-cell recordings in both cells are obtained, and spikes are induced via brief repetitive stimuli to measure the evoked EPSP/IPSP amplitudes and the direction of the connection(s) [13]. Whole-cell recordings from up to twelve simultaneously recorded neurons have been used to study the organization of local connectivity in brain slices [3, 13]. Overall, the data obtained from patch-clamp recordings have provided essential information on the mechanisms underlying neuronal circuit computation, that, so far, could not be provided by EM-based reconstructions. This holds particularly for patch-clamp studies that were performed *in vivo* [28], which do not suffer from potential slicing artifacts [27]. Despite attempts to automate and scale up patch clamping procedures [29], the throughput for connectivity studies has remained comparably low.

Besides inferring synaptic connectivity from intracellular recordings, there has been a surge in studies that used the statistical relationship of the activity among neurons as an indirect measure of neuronal coupling [30]. Spike train cross-correlograms (CCGs), for example, have been applied to estimate spike transmission or effective connectivity between defined neurons and/or specific brain regions [31–36]. To improve the performance of CCG-based circuit inference, several modifications have been suggested—such as, to take into account co-modulating background dynamics [37–39], to apply model-based timescale separation techniques [40, 41] or, more recently, to apply deep learning methods [42]. Still, inferring synaptic connectivity from the ongoing spiking activity of neurons remains highly challenging [43]. This holds, in particular, if the strength of a synaptic connection is weak [40], if the neuronal networks cannot be fully sampled [44], and if the used spike trains exhibit strong temporal periodicity, e.g., caused by correlated network bursts [39, 45]. Such burst activity may lead to high spike train synchronicity between two neurons that, however, are not synaptically connected [45]. This limits the interpretability of CCG-based methods but also holds for other algorithms used to infer interneuronal coupling in neuronal networks, recorded with either electrophysiological [30] or optical methods [46, 47]. It is important to be aware of these caveats when interpreting the topology of neuronal networks obtained from such activity-based connectivity-inference methods [48–52].

In this study, we introduce a workflow to benchmark algorithms that have been applied to infer neuronal connectivity from large-scale extracellular recordings [36, 40, 53–56]. We, therefore, standardize the output of algorithms and compare statistically inferred connectivity estimates on synthetic ground-truth data sets and experimentally obtained connectivity labels. The first ground-truth data set was generated by stimulating leaky integrate-and-fire (LIF) neurons with empirical spike-train data and statistically defined noise [41]. These data facilitated the study of how changes in network dynamics and the duration of recordings impact network reconstruction performance. In addition, we evaluated the effectiveness of connectivity-inference algorithms using *in vitro* data, with connectivity labels derived from simultaneous high-density microelectrode array (HD-MEA) / patch-clamp recordings obtained from primary neuronal cultures [57, 58]. Finally, we introduce an *ensemble artificial neuronal network* (eANN) and probe whether ensemble learning techniques [59] can improve today’s methodology to infer synaptic connectivity. We train the eANN on the standardized output of several existing network-inference methods and demonstrate that knowledge about the shared output from these methods does indeed lead to gains in network reconstruction performance. To this end, we use a SHapley Additive exPlanations (SHAP) [60] analysis to better understand the relative contribution of individual input features to the superior eANN performance, and to visualize and interpret which methods were driving the eANN model to predict excitatory or inhibitory synaptic connections.

## Results

### A framework for the systematic comparison of activity-based connectivity inference algorithms

**Fig 1:**
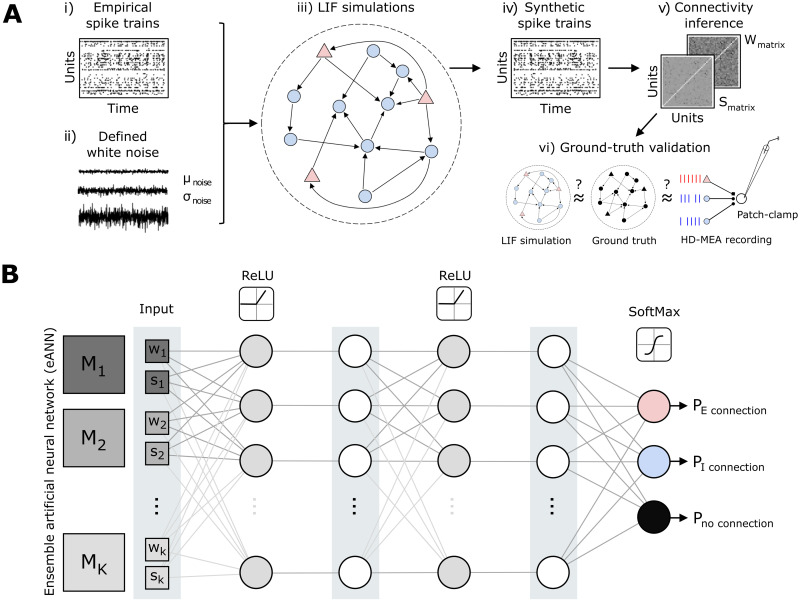
An ensemble artificial neural network to improve neuronal connectivity inference. **A** Schematic illustrating the developed analysis workflow to systematically compare statistically derived neuronal connectivity across inference algorithms and defined network dynamics. Empirical spike-train data (i), obtained by high-density microelectrode array (HD-MEA) recordings from primary cortical cultures, and different types of white noise (ii) were used as input to a network (iii) of leaky integrate-and-fire (LIF) neurons (300 neurons, 50:50 excitatory (E) and inhibitory (I) neurons), adopted from previous work [41]. The *a priori* defined structure underlying the LIF network served as the first ground truth to compare established and new connectivity-inference methods, providing a connectivity score (*s*) and a weight (*w*) for each connection (v-vi). Moreover, connectivity-inference performance was also assessed on connectivity data obtained from parallel HD-MEA/patch-clamp recordings [58]. **B** Schematic depicting the architecture of the ensemble artificial neural network (eANN). The eANN receives as input the connectivity score *s* and weight *w* values from multiple established inference algorithms. Then the feed-forward network is trained. Finally, the eANN outputs probability values which indicate whether the connection is excitatory, inhibitory, or if there is no connection at all.

To compare inference algorithms and to evaluate their performance, we standardized the connectivity inference task: Each inference method received the same spike-train activity, i.e., the spike times and the corresponding unit IDs of a given neuronal network recording. These data consisted of either a network simulation (see Sec A in S1 Text; Fig 1A), or an HD-MEA extracellular network recording obtained from primary cortical cultures (see Sec C3 in S1 Text). Then, as output, each connectivity inference method provided two results: (i) a directed weighted graph, that indicated the connectivity strength between all units, and (ii), a matrix that contained the *connectivity scores* for all connections. We will refer to the first output as the *weight graph* (W), and to the second output matrix as the *score graph* (S). Each edge of the inferred connectivity score graph S represents a connectivity score.

For a given putative connection between a presynaptic neuron *i* and postsynaptic neuron *j*, we defined a connectivity score *s*_*i*→*j*_. The connectivity score indicates the likelihood of a putative monosynaptic connection according to the respective inference method. Of note, the meaning of the connectivity score depends on the method: For example, the connectivity score could be a (log) probability, a negative (log) p-value, or the absolute value of a z-score (see Sec E in S1 Text for the definition of connectivity and Table 1 for an overview on all implemented methods). As for the connectivity score graph *S*, the interpretation of the weight graph *W* depends on the respective inference method, e.g., it might indicate an estimate of synaptic strength or how much information about the spike train *j* is contained in spike train *i*. For a given connection from neuron *i* to *j*, we define a weight *w*_*i*→*j*_.

**Table 1:** Overview of connectivity inference methods and abbreviations.

Method name	Abbreviation	Description
Coincidence index	CI	Measures the excess/lack of coincidental firing compared to what would be expected by chance (shuffled data). Adapted from [56].
Smoothed cross-correlogram	sCCG	Quantifies the temporal correlation between two spike trains; a smoothing kernel is used to discern background activity from synaptic interactions [37].
Directed spike time tiling coefficient	dSTTC	Quantifies the synchrony between two neurons by considering the temporal overlap and rate of spikes in a defined temporal window [53].
Generalized linear model (GLM) cross-correlogram	GLMCC	Parameterized generalized linear model to facilitate synaptic connectivity inference from cross-correlograms [40].
Transfer entropy	TE	Measures how knowledge about the spike history of one neuron reduces the uncertainty about the future spiking of another neuron [54, 65].
Generalized linear model (GLM) point process	GLMPP	Generalized linear model to parametrize firing rate according to network history. Modified from [72] and combined with methods from [73].
Ensemble artificial neural network	eANN	Neural network trained on CI, sCCG, dSTTC, GLMCC, TE and GLMPP to predict connectivity, as introduced in this study.

We consider a putative connection between neuron *i* and *j* as present when the respective connectivity score *s*_*i*→*j*_ is larger than a statistically defined threshold. This threshold, however, has to be defined by the experimenter. Connections below this defined threshold are regarded as unconnected pairs. As synaptic connectivity is generally assumed to be sparse, this task is highly unbalanced, i.e., we would expect that the unconnected pairs largely outnumber actual connections. Such imbalance poses a problem for accuracy measurements [61]. To compare network reconstruction performance across algorithms, and to take this imbalance into account, we here calculated the averaged precision score (APS). The APS is defined as the integral of the precision-recall curve, which attains values between 1 (the best possible outcome) and 0 (the worst outcome). Since the APS is an aggregate statistic over all possible thresholds and some analytical questions do require thresholded graphs, we considered the Matthews correlation coefficient (MCC) [62] as a second performance metric. See Sec F in S1 Text for details.

### Overview on connectivity inference algorithms

Historically, many studies have applied cross-correlograms (CCGs) to estimate putative mono-synaptic connections between neurons [31–33, 35–37]. In the present study, we considered three CCG-based connectivity inference methods: First, the *coincidence index* (CI), which integrates the CCG over a small synaptic window [56] and compares it to values obtained from surrogate data (i.e., jittered spike trains for which the short-latency synaptic relationships have been destroyed). The second method convolves CCGs with a partially hollow Gaussian kernel [37], and thereby allows separating slower background activity from faster synaptic interactions. We refer to this method as the *smoothed cross-correlogram* (sCCG) algorithm [36]. The third CCG method, finally, fits a *generalized linear model* (GLM) to the spike-train CCGs [40]. As in the original publication [40], we refer to this method as the GLMCC algorithm. The GLM models the background spiking and the potential synaptic effect as two separate additive functions: The stronger the contribution of the synaptic effect is, the more likely a synaptic connection.

Several studies have applied information-theoretic measures to estimate neuronal connectivity and information flow between brain regions and individual cells [51, 52, 54, 63, 64]. Here, we utilized an efficient algorithmic implementation of *transfer entropy* (TE) [65] to infer connectivity from discretized spike trains. TE has been used as a measure of information flow and quantifies—in this context—if information about the spike activity of neuron *j* improves the prediction of the activity of neuron *i* in addition to knowledge about the spiking history of neuron *i* alone [64].

We also implemented a modified, directed variant of the *spike time tiling coefficient* (dSTTC) [53]. Although originally not developed to quantify synaptic connectivity, but rather the synchronicity between pairs of spike trains, it has recently gained a lot of popularity [66–68]. The dSTTC variant implemented here quantifies interneuronal coupling by estimating the proportion of spikes of two units that appear within a specific synaptic window. More specifically, the dSTTC estimates whether there is an excess or scarcity of spikes of neuron *j* following the spikes of neuron *i* as well as spikes of neuron *i* that were elicited before those of neuron *j*.

Finally, the last inference method was based on a *generalized linear model for point-processes* (GLMPP) [55]. The GLMPP model allows the integration of multiple covariates, such as the past activity of the entire network, in an explanation of the observed spiking activity of a neuron *i*. Models of this class have been used in the past for inferring connectivity among partially observed neuronal populations [69].

Each inference method was adapted to the previously described analysis framework, i.e., each method provided a *score graph* S and a *weight graph* W. For more details on all implemented algorithms, and the applied modifications to fit them to our workflow, see Sec E in S1 Text. An overview of all methods is provided in Table 1.

### Using an ensemble artificial neural network to improve connectivity inference from spike trains

Due to the great diversity of neuronal cell types and connectivity, and the inherent complexity of neuronal dynamics, it is likely that network-reconstruction algorithms perform better in some scenarios than in others [46, 70]. In this study, we introduce an algorithm that is based on an *ensemble artificial neural network* (eANN) which allows for the prediction of synaptic connections. This capability is achieved after training the eANN on ground-truth simulations and the corresponding output of several existing inference methods (see previous section). Hence, one goal was to probe whether the collective input from traditional inference algorithms—as learned by the eANN—improves network reconstruction accuracy and robustness (Fig 1).

To address this question, we first trained the eANN on connectivity-inference results derived with conventional algorithms from *leaky integrate-and-fire* (LIF) network simulations (see Sec A in S1 Text). For a putative connection between neurons *i* and *j*, the resulting eANN received the weight *w*_*i*→*j*_ and connectivity scores *s*_*i*→*j*_ of all six inference methods as input. The implemented eANN architecture consists of a feed-forward network [71], with two hidden layers with ten units each, and a rectified linear unit (ReLU) non-linearity. The output layer has three units and a softmax non-linearity, and indicated either an excitatory (E) connection, an inhibitory (I) connection, or no connection at all (Fig 1B). Since we hypothesized that excitatory and inhibitory connections might be reflected differently by each method, we chose a multi-class classification setting. The model was then trained on different LIF simulations by minimizing the cross-entropy loss using the ground-truth labels in the training data. The final connectivity score *s* of the eANN was defined as the maximum softmax output for either an excitatory or inhibitory connection. If the connectivity score for a pair *i* → *j* exceeded a specific predefined threshold, the connection was deemed significant. The eANN method only predicts if a connection exists or not. Although we found that the connectivity score *s* is correlated with the simulated synaptic strength (see Fig H in S1 Text), we leave the prediction of actual synaptic strengths to future research. Of note, the eANN is only trained once on several dynamical regimes (see Table A in S1 Text), and all subsequent results were obtained from this single instance of training.

### Comparing network reconstruction performance across algorithms, network dynamics, cell types, and recording duration

**Fig 2:**
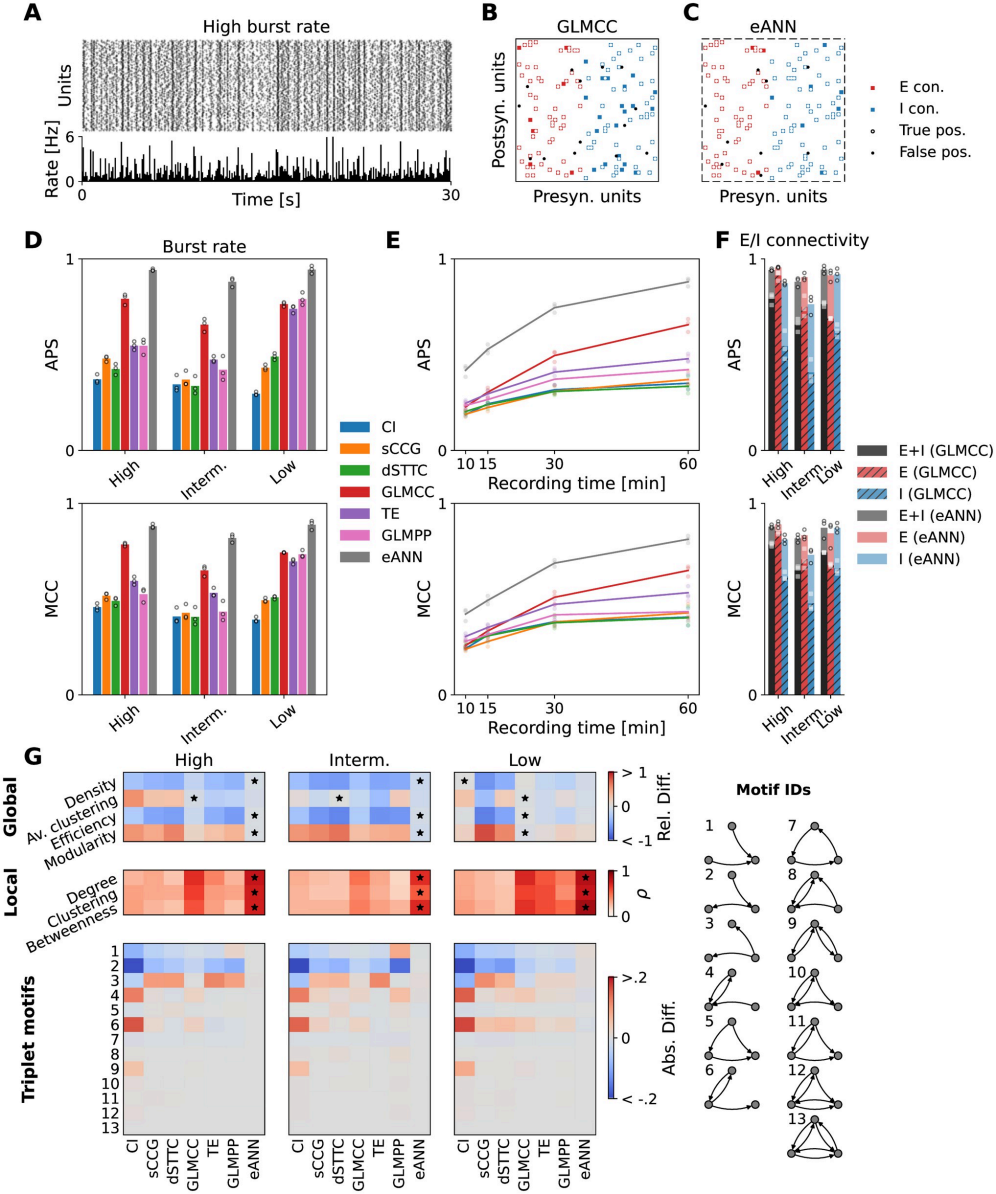
Reconstruction performance across algorithms, dynamics, cell types, and recording length. **A** Example raster plot (upper panel) and traces of binned population activity (lower panel; the number of spikes per second and neuron) of the *high-burst rate* condition. **B** Network reconstruction obtained from a subset of the data shown in **A**, exemplified for the GLMCC method [40]. Red and blue squares correspond to ground-truth excitatory and inhibitory synapses. White and black circles indicate predicted true positives and false positives. **C** Same as **B**, for the results obtained with the eANN approach, which generally improved the reconstruction performance. **D** The mean average precision score (APS, upper panel) and Matthews correlation coefficient (MCC, lower panel), estimated across all connections, obtained from all inference algorithms and the eANN across three different dynamical regimes. Dots depict the performance obtained on three different subnetworks of the same simulation. **E** Connectivity reconstruction performance (APS, MCC) as a function of recording time. Results indicated an improvement for longer recordings. Results in panel **E** are depicted for the intermediate dynamical regime. **F** APS and MCC for each type of connectivity, i.e., either excitatory (E, in red), or inhibitory (I, in blue), or a combination of both types (E+I, in black). Correspondingly, the performance gains achieved by the eANN are plotted in shades of red, blue, and black. **G** Quality of topological feature reconstruction for the inferred network across the three dynamical regimes. In the upper panel, the relative difference between four global features (network density, average clustering, and efficiency) is shown. The panel in the middle shows average Pearson correlation coefficients for the local/nodal metrics, comparing values obtained for the ground truth and inferred networks. Black stars indicate the method that performed best. The lower panels depict the absolute difference of triplet-motif frequencies between the ground truth and the inferred networks.

To benchmark inference methods, we first turned to LIF simulations (see Sec A in S1 Text for details), which were obtained similarly to an approach outlined in a previous study [41]. The spike-train output of the LIF simulations resembled the subsequently used *in vitro* HD-MEA recordings. Each LIF network consisted of *N* = 300 neurons, equally split into excitatory and inhibitory neurons, and connected randomly with a probability of 0.05. The LIF network received two types of input: i) spike-train data from an experimental recording to mimic realistic dynamics (connection probability to LIF neurons: 0.1), and ii) white noise input. The white noise was varied, to map out a range of different dynamical regimes. As benchmarking datasets, we generated three different simulations: a high-bursting regime (burst rate: 1 Hz, average firing rate: 1.6 Hz, coefficient of variation: 0.3, see Fig 2A), an intermediate-bursting regime (0.4 Hz, 1.1 Hz, 0.4), and a low-bursting regime (0.2 Hz, 1.3 Hz, 0.2). To test the robustness of our algorithms, we grouped each simulated network dataset into three subsets, each composed of 100 neurons (50 excitatory and 50 inhibitory neurons); each simulated dataset was 60 min long (see Fig G in S1 Text, for results obtained for different compositions of excitatory/inhibitory neurons).

In Fig 2, we provide an overview of the benchmarking results for all implemented inference algorithms, including the eANN, across different activity regimes, excitatory and inhibitory connectivity types, as well as network inference calculations run on fewer data (subsets of 10, 15 or 30 min of the data). Example activity for the simulated high-burst regime is depicted in Fig 2A. A network reconstructed by two of the best-performing algorithms (GLMCC and eANN) is shown in Fig 2B and 2C; the average APS and MCC statistics across all methods are depicted in **D**-**E**); the reconstruction performance for excitatory and inhibitory connectivity is shown in panel Fig 2F.

In line with previous research [43, 46], we found that the network reconstruction from ongoing spiking activity was altered, if the provided spike trains contained periods of very synchronous activity. This becomes also apparent for the results obtained by the GLMPP and TE algorithms (see Fig 2D). While the performance of these algorithms was good for the low-burst regime (e.g., TE: APS = 0.74, MCC = 0.70; recording duration: 60 min; connectivity thresholded at maximum MCC value), it deteriorated strongly for recordings of the high-burst regime (TE: APS = 0.55, MCC = 0.59). Here, the inferred connectivity suffered from many false positives. The performance for the GLMCC method seemed less affected (GLMCC: APS = 0.76 (low)/0.79 (high), MCC = 0.74/0.79). The performance of the GLMCC algorithm for excitatory connections was fairly good overall (Fig 2B; excitatory connections in red), but less robust for inhibitory connections (few false positives; Fig 2B and 2F; inhibitory connections in blue). For all methods, reconstruction performance decreased for shorter recording lengths (Fig 2E), again, in agreement with previous reports [40, 74]. Very similar performance profiles were also observed for most of the other inference algorithms (Fig 2D and 2E). The reconstruction performance of most methods increased for simulated networks with a higher percentage of excitatory neurons, such as an 80:20 (excitatory to inhibitory neuron) ratio (see Fig G in S1 Text).

Next, we probed the network-reconstruction performance of the eANN. Results indicated that the eANN outperformed all other inference methods—both, across all dynamical regimes (Fig 2D), and when applied to shorter recordings, respectively fewer data (Fig 2E). The most substantial improvements were observed for the intermediate-burst regime. Here, the average APS for the eANN was 0.88, i.e., a 35% improvement compared to the best-performing model (GLMCC: APS = 0.65). Similarly, we found a 24% improvement in the MCC values for the eANN (eANN: MCC = 0.81) compared to the GLMCC (eANN: MCC = 0.65; see Fig 2D). The main performance gains for this condition resulted from a reduction in false positive excitatory connections and an increase in true positive inhibitory connections (see Fig 2B and 2C). For the temporal subsampling analysis (Fig 2E), we compared the reconstruction performance of all algorithms on 10, 15, 30, and 60-minute subsets of the data. Although the eANN performance also decayed for shorter data segments, it was still significantly better compared to the other methods. Interestingly, the accuracy values of the best-performing traditional inference method, the GLMCC algorithm, decreased strongest for shorter recordings (lowest ASP/MCC values for spike-train data below 30 minutes); see Fig A in S1 Text for the precision-recall curves across all datasets and algorithms.

Finally, we probed how accurately the different algorithms could infer the global and local topological statistics of the simulated ground-truth networks. For details on the different topological statistics see Sec D in S1 Text. For the global metrics, we quantified the dissimilarity between the inferred networks *F*_inf_ and the ground-truth networks *F*_gt_ by their relative difference (*F*_inf_ − *F*_gt_)/*F*_gt_. For the local topological metrics, we computed the Pearson correlation *ρ* between all nodal values of the inferred network and the nodal values of the ground-truth network; the reported values were averaged over three different networks. In Fig 2G, we report summary results for reconstructed graphs that were binarized with the best-performing MCC value. We found that many of the inferred connectivity metrics, including basic properties such as the network density, but also nodal features (e.g., the average clustering coefficient), differed substantially between algorithms and dynamical regimes. As expected from the results depicted in Fig 2D, most connectivity methods, and in particular the GLMCC algorithm, performed fairly well in the low-burst regime. Here, we found only little relative differences between the global/local topology of the ground-truth networks and the topology of the inferred networks. For the intermediate and high-burst regimes, however, the estimates became worse. Here, the eANN excelled and outperformed all other methods. The average relative difference in global topological metrics for the eANN was 4%, 10%, and 6% for the low, intermediate, and high-burst regimes, respectively. For the second-best method, the GLMCC algorithm, the relative difference in global topological metrics were 2% (low), 27% (intermediate), and 13% (high-burst regime). For the local features, the eANN performed best on average (Pearson’s correlation: 0.74, 0.63, 0.81 for the high, intermediate, and low-burst regime); the performance of the GLMCC algorithm was still good, but lower on average: 0.62 (high), 0.44 (intermediate), and 0.65 (low-burst regime). The good reconstruction performance of the eANN algorithm became even more apparent for the inference of triplet motifs [18]. While many algorithms showed similar results in their estimates of the frequency of motifs, the eANN performed overall very well (Fig 2D). We also characterized the topology of graphs binarized with an absolute threshold, as derived from jittered surrogate spike-train data (*α* = 0.01; Fig B in S1 Text). In line with the results presented here, the eANN showed again robust performance.

### SHAP analysis of eANN output

**Fig 3:**
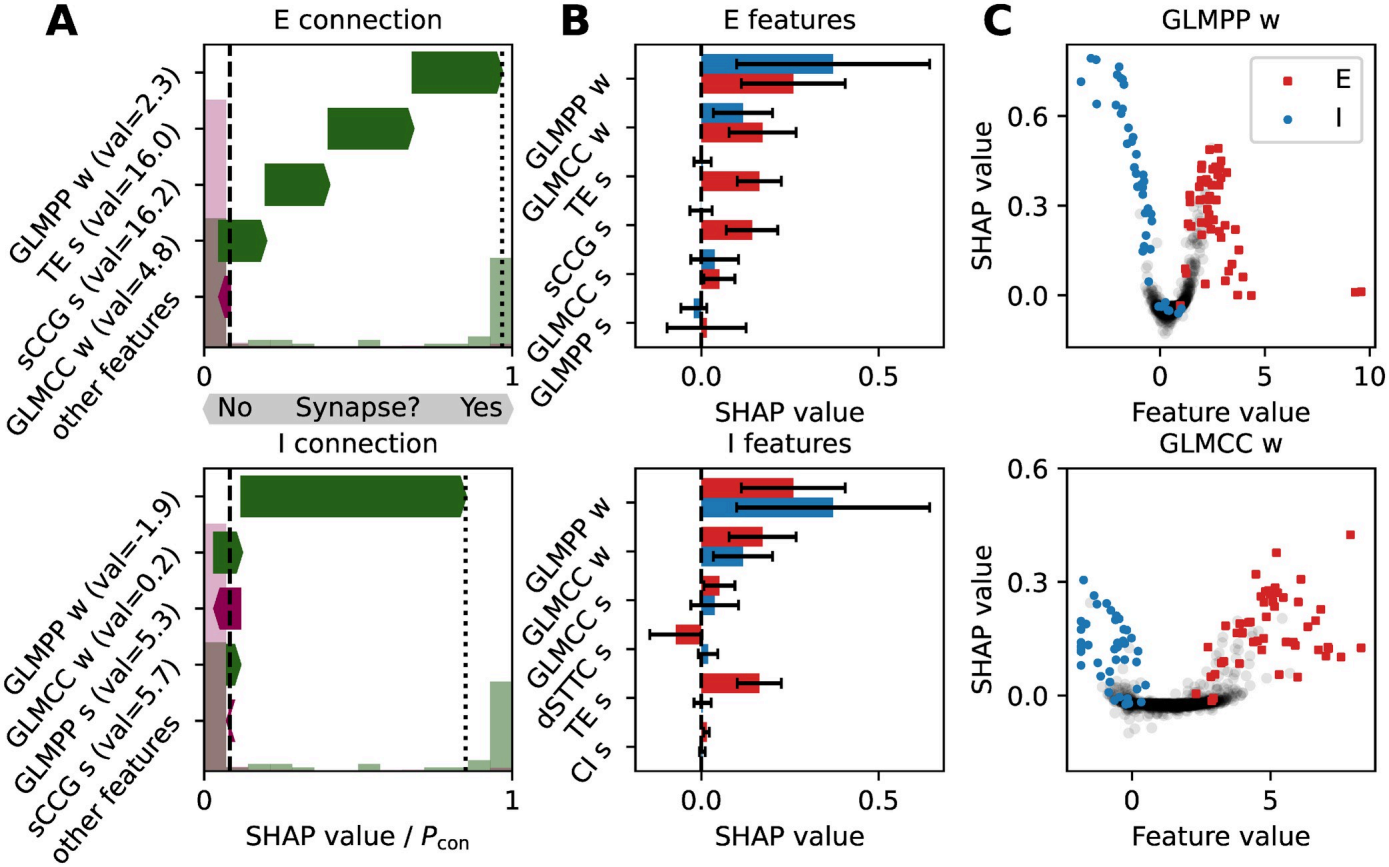
SHAP analysis on eANN output. **A** SHAP values for an examplary excitatory (E) connection (top panel) and an inhibitory (I) connection (bottom panel), ranked according to their importance. The length of the arrows denotes the approximated feature contribution to the eANN output, as estimated by the SHAP method [60]. The y-axis shows the corresponding features with the feature value in brackets. Green arrows pointing to the right indicate that this feature was informative in the process of determining an E/I connection; purple arrows pointing to the left indicate the opposite. Normalized histograms plotted in light green and purple show the eANN output for connected pairs and unconnected pairs. The dashed line is the average eANN output for all connections in the dataset. **B** The features with the highest average SHAP values for excitatory (E, top panel) and inhibitory (I, bottom panel) connections. Red and blue bars depict the average SHAP values for excitatory and inhibitory connections; error bars represent the standard deviation, calculated over all E/I connections present in the dataset. **C** SHAP values as a function of feature values for the top two features depicted in panel **B**, namely GLMPP *w*, and GLMCC *w*; black dots indicate unconnected pairs.

Next, we sought to understand in more detail which input features of the eANN contributed most to the improved reconstruction performance. We, therefore, investigated the eANN output with a SHAP analysis [60]. A SHAP analysis allows determining how much each of the connectivity methods (i.e., the input features) adds to the eANN predictions of excitatory or inhibitory connections. The SHAP values, which are calculated for each of the connectivity methods and each prediction, represent the contribution of each method to the prediction task—and thereby explain partly the inner workings of the eANN model. Fig 3 depicts the SHAP values for 1500 putative connections obtained from an eANN analysis performed on simulated spike trains in the intermediate-burst regime. In panel Fig 3A, we depict SHAP values for example inhibitory and excitatory connections. The results indicate that approximately three features contributed most prominently to the decision of the eANN in favor of an excitatory (E) connection (upper panel), namely, the obtained weight of the GLMPP method and the connectivity score values of the TE and sCCG algorithms. For the inhibitory (I) connection (lower panel) mainly the weights of GLMPP and GLMCC methods contributed to the decision. In Fig 3A the lenght of the arrows indicate contribution to the eANN output. Green arrows depict positive and purple arrows negative contributions. While many features seemed to carry information about excitatory connections (Fig 3B, top panel) only about two features were predictive for inhibitory connections. However, the SHAP values for these features were quite variable. Interestingly, we observed that the features that yielded—on average—the highest SHAP values, were the same for excitatory and inhibitory connections (i.e., the weight estimates inferred by the GLMCC and GLMPP algorithms). However, we also found differences in how features contributed to detecting excitatory and inhibitory connections. For example, while the connectivity score of the sCCG method contributed to the detection of excitatory connections, it contained only little information for the inference of inhibitory connections. To illustrate how single features contribute to the classification of excitatory/inhibitory connections, we depict the exact SHAP values of the two top-ranked metrics (GLMPP and GLMCC) as a function of their feature values (Fig 3C): Results indicate that the two features were mainly negative for inhibitory connections (depicted in blue) and positive for excitatory connections (depicted in red); high SHAP values often correlated with large absolute feature values. This held, in particular, for the negative weight estimates of the GLMPP, indicating that this feature may contribute strongly to the identification of inhibitory connections. It is noteworthy, that none of these features alone was sufficient to separate actual connections from unconnected pairs. We propose this underscores that the eANN approach is advantageous for the reliable inference of connectivity from spike-train data. Finally, we investigated the correlations among input features (Fig K in S1 Text). Interestingly, we find that features indicative of excitatory connections are much stronger correlated than features for inhibitory connections. This indicates that different methods may carry different kinds of information, and that the eANN can utilize this information for the prediction of inhibitory connections.

### Application to in vitro data obtained by parallel HD-MEA/patch-clamp recordings

**Fig 4:**
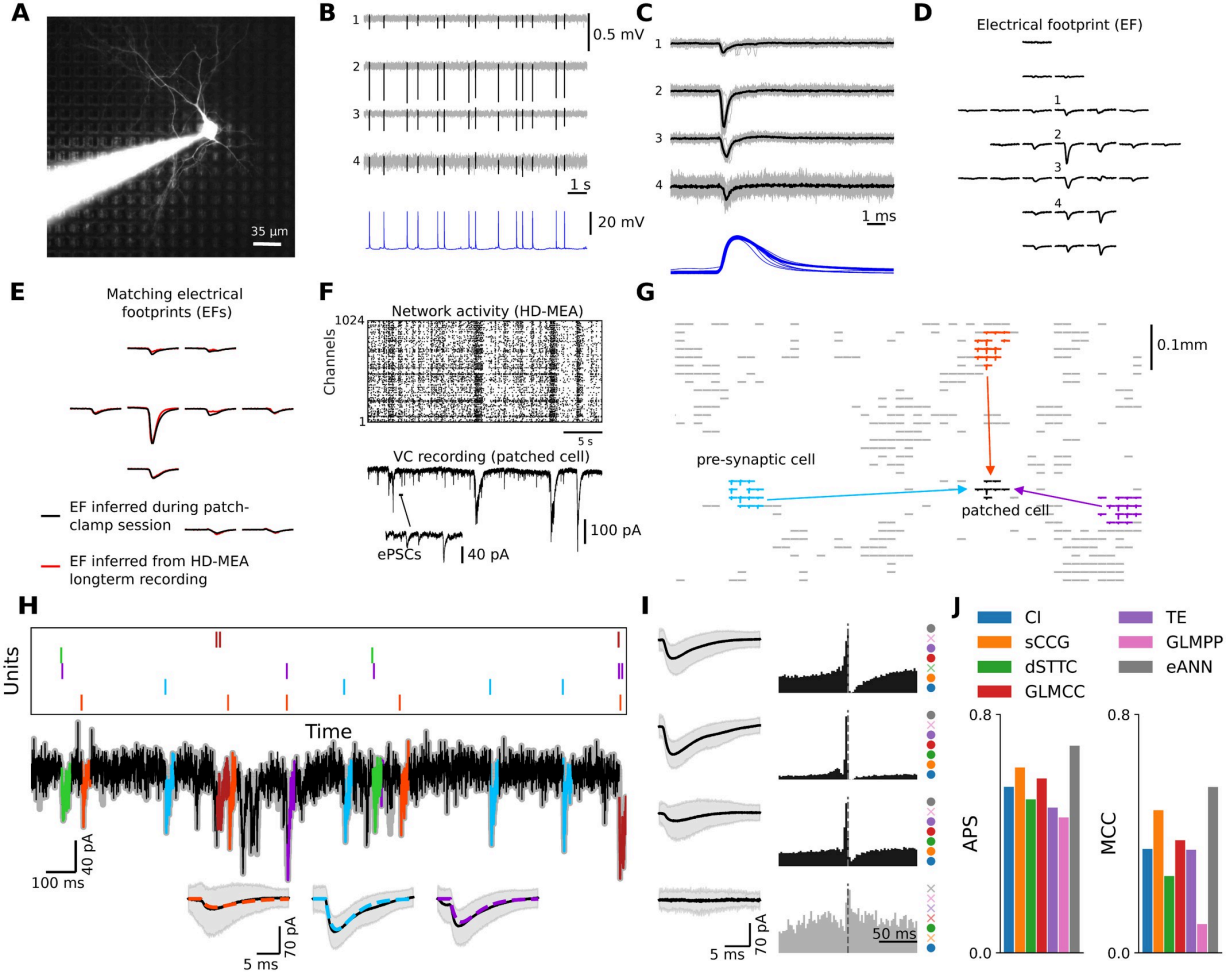
Validating synaptic connectivity inference with parallel HD-MEA/patch-clamp recordings. **A** A single patched neuron on the HD-MEA, including the patch pipette. The neuron was labelled with Alexa Fluor 594 through the patch pipette; HD-MEA electrodes can be seen in the background. **B** Example recording of a patched neuron (lower panel, intracellular signal in blue), and some of its extracellular signals on the HD-MEA (upper panel, spikes in black); HD-MEA and patch-clamp recordings were performed in parallel and later synchronized. **C** The temporally aligned HD-MEA/patch-clamp signals allowed inference of the exact location of patched neurons on the HD-MEA and their electrical footprints (EFs) **D**. To infer the putative pre-synaptic connectivity of individual patched neurons, we first performed long-term HD-MEA baseline recordings. Next, we applied a post-processing step to match the EF of the patched cell with the EF templates obtained from the spike-sorted HD-MEA baseline recording. Panel **E** depicts the overlap of the EF generated during the patch session (in black), and the matched EF obtained from the spike-sorted baseline recording (in red). **F** HD-MEA network recording (upper panel) and simultaneous whole-cell voltage-clamp (VC) recording (lower panel). VC recordings were used to measure the excitatory/inhibitory postsynaptic currents (ePSCs/iPSCs) in the patched cell, and to link their occurrence to the extracellular activity of neurons recorded on the HD-MEA. Panel **G** depicts three exemplary connections of the patched cell (EF in black) to three presynaptic neurons; the EFs of these cells are depicted in light blue, orange, and purple. **H** IC model fit to patch-clamp recording. Spikes of identified presynaptic neurons are shown on top, aligned with the recorded currents of the patched neuron. PSCs of three units are shown as insets, with the average PSC signal depicted in black and the fitted model PSC displayed in different colors. The shaded area shows the 5–95% quantile range. **I** PSCs of four cells, three connected presynaptic neurons, and one unconnected neuron, are shown along with their corresponding CCGs. Note, that a high-chloride internal patch-clamp solution was used to simultaneously measure the synaptic activity of excitatory and inhibitory presynaptic cells [58]. As a result, as depicted, inhibitory input currents also have a negative polarity. On the right side of the CCG, a colored circle indicates whether the respective connectivity method found a connection or not (cross). **J** Panel depicts the performance of the different connectivity methods averaged over three patched neurons.

Next, we applied the developed connectivity-inference pipeline to empirical data, obtained through parallel HD-MEA/patch-clamp recordings from *in vitro* neuronal cultures (dataset 1; for details on the data see [58]; Fig 4; n = 3 patched cells; culture age: DIV (days in vitro) 17–18; see Sec C2 in S1 Text for details). Briefly, following an HD-MEA baseline recording for spike-train-based connectivity inference (∼3 h long), neuronal cultures were transferred to a setup that allowed for simultaneous HD-MEA/patch-clamp measurements. Here, single neurons were patched on the HD-MEA and, in parallel, recorded extracellularly on the HD-MEA. Two distinct HD-MEA/patch-clamp recordings were obtained from each patched cell. First, we recorded spontaneous spiking of the patched cell in whole-cell current-clamp mode in addition to simultaneously recording extracellular HD-MEA signals. The obtained data then allowed to perform spike-triggered averaging (Fig 4A–4E) and to infer the *electrical footprint* (EF) of the patched neuron. The EF is the extracellular profile of a neuron on the HD-MEA (Fig 4D and 4E).

To study the synaptic input to the individual postsynaptic cells during ongoing spontaneous activity, we recorded neurons in voltage-clamp mode (Fig 4F). As previously reported [57, 58], combining the spontaneous/evoked electrical activity of neuronal networks, recorded on the HD-MEA (Fig 4F), with the measured postsynaptic currents (PSCs) in patched neurons, allows reconstructing connectivity to putative presynaptic partner cells. To estimate the effect of extracellularly recorded neurons on the patched cell, and most importantly, to infer synaptic connections between the patched neuron and its putative presynaptic network, we developed a regression-based connectivity inference method (similar to [75], see Sec B in S1 Text for details). The method was first benchmarked on *in silico* data; an overview of the obtained performance results is provided in Fig E in S1 Text. Our *in silico* results indicated that the developed method can robustly detect synaptic connections (high recall), with only few false positives (high precision). Although the performance deteriorated for low-rate conditions and more correlated spiking, the method proved very reliable for a wide range of parameters.

Next, we applied the developed intracellular connectivity-inference method to three patch-clamp recordings obtained from a previously published dataset [58], and identified 26 putative synaptic connections among 131 possible combinations. In Fig 4H we show an example fit with three PSC cutouts of three identified connections in an example recording; Fig 4I depicts the CCGs and PSCs of three connections found in the recording and an unconnected pair. For an overview of all PSCs and corresponding pair-wise CCGs of identified connections, as well as for information on which algorithms succeeded in detecting the connection, please see Fig F in S1 Text.

With this labelled experimental data at hand, we then evaluated the performance of the existing activity-based inference methods, and the eANN, on 3-h-long HD-MEA baseline recordings. Of note, the eANN is the same as for Fig 2 and was not re-trained for these analyses. For the *in vitro* data, we see similar performance for the six input methods in terms of APS, but varying performance for MCC (see Fig 4J). The GLMCC algorithm (MCC = 0.37), the sCCG (MCC = 0.49), and the TE algorithm (MCC = 0.36) performed best among the input methods. The superior performance of GLMCC originates rather from higher precision than recall (recall = 14 correctly classified connections of 26 connections; precision = 14 correctly classified connections among 39 identified synapses). In comparison, the sCCG method (recall = 23/26; precision = 23/53) and the TE algorithm (recall = 16/26; precision = 16/38) both yielded similar or higher recall, at the expense of lower precision. As for the analysis with simulated ground-truth data, we found that the eANN (MCC = 0.56) outperformed the other methods in terms of both MCC and APS. Again, the dominating factor was, that the eANN yielded much higher precision at inferior recall (recall = 17/26; precision = 17/26). Considering these numbers, in particular, for recall and precision, we found that this performance increase was mainly due to more accurate predictions (higher precision), at the cost of not identifying some true connections (lower recall). In summary, our results indicate that connectivity inferred by the eANN method is more precise than the results obtained with any of the other implemented inference methods.

### Characterizing in vitro neuronal network connectivity and topology

**Fig 5:**
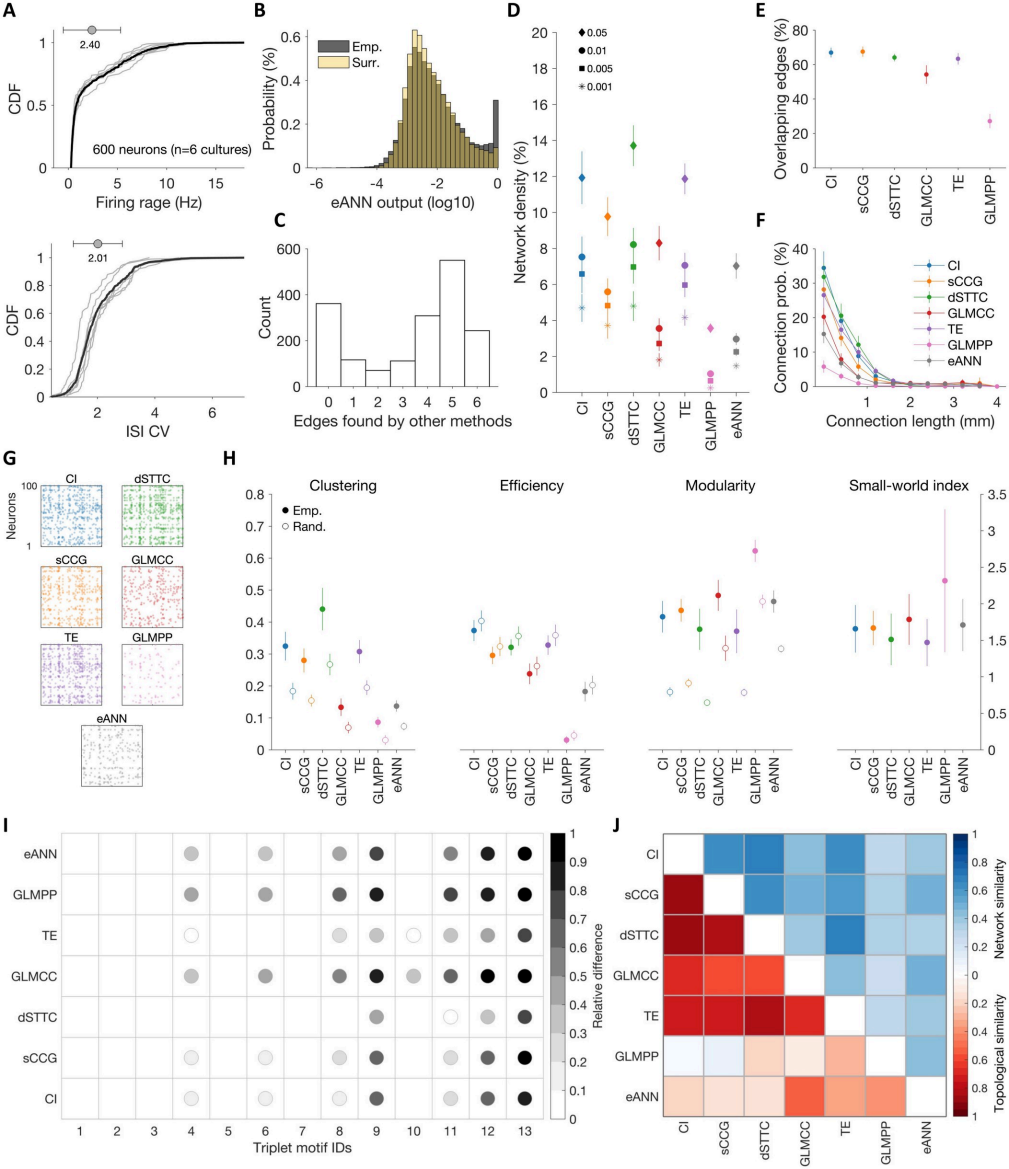
Characterizing the connectivity of in vitro neuronal networks. **A** Firing rate cumulative density function (CDF) of 600 spike-sorted units from HD-MEA recordings obtained from primary cortical cultures (top panel; n = 6 cultures; 100 units per network; recording duration: 1 h; culture age: DIV 14); and CDF of inter-spike interval coefficients of variation (ISI CV) for the same cultures (lower panel). **B** Overall distribution of eANN weights of empirical networks (in gray; values are depicted in logarithmic space) and overlaid with the corresponding distribution of eANN values inferred from surrogate networks (in yellow). The distribution of experimentally inferred values demonstrates a clear peak in the eANN weight distribution that distinguishes putative synaptic connections from unconnected pairs. **C** Significant eANN edges cannot exclusively be explained by the overlap across all inference methods. Panel E depicts the consensus distribution, i.e., the overlap across the six inference methods for all significant eANN edges; most eANN edges were found by five of the other methods. About 20% of edges were found by the eANN, but not by the other methods at the selected threshold (see zero bin). **D** Network density as a function of threshold values (corresponding to *α*: 0.05, 0.01, 0.005, and 0.001) across all inference methods. *α* threshold values were derived from surrogate connectivity estimates (temporally jittered spike trains). Network density decreased with smaller *α*-values, and varied significantly across methods. **E** Intersection of significant eANN connections with those of all other connectivity inference methods. **F** Inferred connectivity decayed with interneuronal distance (*α* = 0.01), and the likelihood of long-range connections (> 1 mm) was very low. **G** Example connectivity matrices for one culture inferred with all seven inference methods. **H** Topology of inferred in vitro networks differed significantly across inference methods (*α* = 0.01; filled circles: empirical data; empty circles: randomized surrogate networks). **I** All inference methods yielded an over-representation of triplet motifs (see Fig 2 for motif ID legend), but with slight differences across methods. Dots depict motif IDs that occurred significantly more frequently than re-wired surrogate networks (FDR corrected *α* of 0.001); the color indicates the relative mean difference in their occurrence. **J** Lower triangle (red color scale): Topological similarity, calculated by pairwise Pearson correlation coefficients across the topological metrics shown in **H** and **I** across all inference methods. Upper triangle (blue color scale): Network similarity, quantified by pairwise MCCs across all inferred adjacency matrices. Panels D-J depict network graphs thresholded with *α* = 0.01.

Finally, we applied the eANN pipeline to a second dataset of HD-MEA network recordings, obtained from primary cortical cultures (dataset 2; n = 6 cultures; recording duration: 1 h; culture age: DIV 14). The main goal of this analysis was to compare how connectivity and topological properties varied across the implemented inference methods (Fig 5). As before, all network recordings were first spike-sorted and underwent several quality-control steps to ensure that connections were estimated on sufficient activity (Fig 5A, see Sec C4 in S1 Text). To reduce between-network variability, we only sampled 100 units from each network.

Comparing the overall distribution of empirical eANN scores (Fig 5B, depicted in black) to the corresponding surrogate distribution of eANN output values (depicted in yellow) indicated that the spike-train jittering effectively destroyed short-latency synchronization between individual neurons. The distribution of experimentally inferred values showed a clear peak in the eANN weight distribution that distinguished putative synaptic connections from unconnected pairs (Fig 5B). Next, we calculated the consensus distribution (Fig 5C), i.e., the overlap between all significant connections obtained by the six traditional inference methods and the network inferred by the eANN (threshold value: *α*=0.01). The results indicated that most eANN edges were found by five of the six inference methods (31.3%). Interestingly, some edges were found by the eANN method, but not by the other methods at the selected threshold (edges in the zero bin: 20.5%). This finding may indicate that some eANN edges could not exclusively be explained by the overlap across all inference methods, but that there is added value by the eANN algorithm at this threshold. The network density decreased with smaller *α*-values (Fig 5D; *α*-values: 0.05, 0.01, 0.005 and 0.001), and varied significantly as a function of inference algorithms (the repeated measures’ analysis of variance (ANOVA) for a *α* threshold value of 0.01 was: F(6,30)=230.31, p = 7.07 × 10^−9^; p-value with Greenhouse-Geisser adjustment); the network density of all graphs was sparse (e.g., for a threshold of *α*=0.01, the network density varied between 1–9%; Fig 5D and 5G), in line with previous reports [18, 76]. All implemented algorithms reconstructed networks that showed a clear decay in connection probability as a function of neuron-to-neuron distance (Fig 5F).

Furthermore, we found that topological properties of network segregation and integration differed as a function of the inference method (clustering: F(6,30)=92.25 p = 1.62 × 10^−7^; efficiency: F(6,30)=277.58, p = 1.70 × 10^−13^; modularity: F(6,30)=30.72, p = 6.19 × 10^−5^; small-world-index: F(6,30)=2.15, p = 0.19; all p-values with Greenhouse-Geisser adjustment; Fig 5H). Despite the reported differences, all algorithms implied that networks possessed a non-random modular, small-world organization (small-world-index > 1); a topological analysis with proportionally thresholded networks is provided in Fig D in S1 Text.

The inferred *in vitro* cortical networks also featured a significant over-representation of some triplet motifs [77] (Fig 5I). The number of motifs that were significantly over-represented in the empirical networks varied between methods (e.g., four motifs for dSTTC and eight motifs for the GLMCC graphs), but all implemented algorithms suggested that motifs 9, 11, 12, and 13 were over-represented compared to appropriate surrogate networks (connectivity threshold: *α*=0.01; motif significance *α*-value = 0.001; p-values were corrected for multiple comparisons across motif IDs and algorithms; Fig 5I). Despite strong differences in the frequency of specific motifs between inference methods, there was a high resemblance across networks within each method (see Fig C in S1 Text). Results obtained from the topology and motif analyses indicated a higher similarity (correlation coefficient) between the output of the eANN and the GLMCC algorithm compared to the other methods (Fig 5J, lower triangle). On average, topological properties and motif frequency values were more similar for networks reconstructed by CI, sCCG, dSTTC, GLMCC, and TE algorithms—as compared to the GLMPP or the eANN. This trend is also reflected in the pair-wise similarity analysis among inferred adjacency matrices, using Matthews correlation coefficients (MCCs) (Fig 5J, upper triangle).

## Discussion

The present study demonstrates that the inference of synaptic connectivity from extracellular spike train dynamics can be improved by the application of an *ensemble artificial neural network* (eANN). By benchmarking the eANN to several existing connectivity-inference algorithms in a standardized analytical framework, we report a superior reconstruction performance for the eANN, that persisted across different dynamical regimes and recording durations. We find that the eANN did also provide a more accurate reconstruction of the type of connectivity (excitatory or inhibitory), and better estimates for the global and local topology of networks *in silico*. Results derived from a SHAP analysis allowed for further validation of the specific contributions of individual algorithms to the eANN performance. Importantly, we found improvements in network reconstruction for both simulated ground-truth data and *in vitro* HD-MEA/patch-clamp data. We propose that such generalizability indicates that the developed method leads to more accurate and robust connectivity inference in datasets for which knowledge of the underlying synaptic connections is not available.

### The challenge to infer synaptic connectivity from spike trains

The extent to which synaptic connectivity and causal relationships between neurons can be studied, based on spike-train dynamics alone, continues to be a matter of debate. Although there have been attempts with small well-established circuits [78, 79], this endeavor has proven challenging for many reasons [43, 80]. In line with previous work [43, 46], the results reported in this study underscore, that there are indeed limits to activity-based network reconstruction (Fig 2D). Using simulations to parametrically model a range of dynamical regimes—we found, as expected, significant performance alterations for most traditional algorithms once spike trains showed stronger temporal periodicity. With few notable exceptions, such as the GLMCC algorithm, reconstruction performance, quantified by the averaged precision score (APS) and Matthews correlation coefficient (MCC), worsened significantly for data with more prominent network burst-activity (Fig 2D).

### eANN improves neuronal network reconstruction

Although ensemble methods have been proposed in the past [59], and used for the study of large-scale brain networks [81, 82], to the best of our knowledge, the present study is the first to demonstrate that such methods can be generalized to cellular spike-train data. More importantly, our results indicate that applying the eANN to such data allowed for significant performance improvements in the reconstruction of synaptic connectivity (Fig 2; Figs A and B in S1 Text). Using a simple feed-forward network architecture [71], and training the eANN on the standardized output of several existing connectivity-inference methods [36, 40, 53–56], we demonstrate that excitatory and inhibitory connectivity, and the topology of neuronal networks, can be reliably inferred from simulated as well as empirical spike-train data (Figs 2 and 4). Overall, the eANN outperformed all traditional methods (Fig 2D–2G), and the performance gains were relatively invariant to different dynamical regimes (Fig 2D) and even persisted on fewer data (Fig 2E). Our results also revealed that the eANN integrates aspects of different inference methods for its connectivity prediction (Figs 3 and 5C), and that high eANN output values were absent, if the millisecond spike-timing information was disrupted, e.g., by jittering the spike trains (Fig 5B). While understanding the performance of neural networks is typically challenging, insights gained from a SHAP analysis indicated that the trained eANN assigned varying levels of importance to different input features, i.e., some inputs were more informative than others (Fig 3). Interestingly, this held in particular for the detection of inhibitory connections (Fig 2F). Here, the GLMPP algorithm seemed to convey valuable input (Fig 3B, lower panel), although it was less effective in the detection of excitatory connections. Overall, results for different connectivity types, network topology, and different network dynamics, as calculated with eANN-inferred graphs, were robust across different threshold definitions (Fig 2, Figs A and B in S1 Text). We hypothesize, that the observed robustness could be attributed to the initial inference methods being designed to be somewhat invariant to differences in firing rates or baseline neuronal activity. Applied to HD-MEA network recordings, eANN-derived connectivity was most similar to graphs inferred by the GLMCC algorithm (Fig 5J and Fig J in S1 Text). Despite these positive results, it should be noted, that the eANN did not result in perfect reconstruction performance. Thus, future studies should probe, whether considering additional features, more completely sampled recordings, or longer recordings could improve network reconstruction even further. Moreover, it would be interesting to probe, if inference performance improves, if data-driven features from other neural network methods, such as the CoNNECT method [42], are integrated into the eANN.

### In vitro neuronal networks show complex topologies

Our results on the putative synaptic connectivity of *in vitro* developing primary neuronal networks, obtained by HD-MEA network recordings, and inferred by the eANN and existing inference methods, are largely in line with previous reports [48, 49, 51, 52, 63, 74]. All algorithms indicated that connectivity was locally clustered, overall sparse (connection probability below 15%; Fig 5D), and that the probability of connections decreased as a function of interneuronal distance (Fig 5F). These results are also in agreement with previous patch-clamp work [13, 76]. Yet, it should be noted, that the effect of the inference method on connectivity and topology was considerable (Fig 5D). For some topological measures, such as the clustering coefficient (Fig 5H), the between-method differences exceeded the differences observed between the empirical and the randomized surrogate networks. For example, the clustering results, calculated on graphs inferred by the eANN and GLMPP methods, were significantly lower than the values of the randomized surrogate networks of some other methods (e.g., CI, TE, and dSTTC). Still, all networks comprised a modular small-world structure (Fig 5H). Some subtle differences in the triplet-motif statistics (Fig 5I) were observed in comparison to previous reports [63], but overall, these were not pronounced, and many of the over-represented motif structures have been reported by whole-cell patch-clamp recordings in slices [13, 18].

### Limitations

Several important limitations should be considered when interpreting the results presented in our study:

First, the analyzed spike-train data were obtained from dissociated primary rodent cortical neurons cultured *in vitro*. Although such model systems have been used extensively to study neuronal physiology at the cellular level [8, 10], and have, thereby, provided fundamental insights into the mechanisms of underlying synapse formation and function, there are limits as to how insights obtained from *in vitro* connectivity can be translated to *in vivo* connectivity data [83]. Future studies should, therefore, train and apply the developed eANN pipeline also to *in vivo* spike-train data—ideally, recorded in well-defined brain subsystems, where inferred connectivity can be linked to structural connections established with other methods [84].

The results presented in Figs 4 and 5 were estimated on DIV 14–18 neuronal networks, a time point where GABAergic signaling may be still immature [85, 86]. Although relatively early during development, our results indicated the presence of some inhibitory connections among the recorded neurons. The relatively young age of these cultures, however, should be considered when interpreting the inferred network density. It is possible that a proportion of synapses is still ‘silent’ at that time, and that some relevant receptors are not yet fully expressed [87]. Also, the cell-plating density of neuronal cultures, respectively the size of networks, affect overall synaptic strengths [76], and hence will impact the ability to infer synaptic connections from activity [88].

A common limitation, shared by all methods in this study, is, that they approximate connectivity from a network that is incompletely sampled. Such subsampling has been shown to lead to altered network reconstruction performance [43, 88, 89]. This holds also true for more complex inference algorithms that can take into account the past activity of the sampled network [54, 69]. Hence, the inferred connectivity may exhibit spurious false positives due to common unobserved input that cannot be explained away [43, 90]. This is also true for the *in vitro* data in our study. Live-cell imaging with calcium or voltage sensors could be applied to improve coverage [46, 70, 91], and future studies should compare how network statistics change as a function of network coverage.

We note, that also the HD-MEA/patch-clamp data used in this study could be further improved [58]. Applying targeted, electrical stimulation to defined pre-synaptic neurons would allow for stronger claims about the found connections and their interneuronal causal effects [57]. Moreover, it would help to have access to the neuritic morphology of some parallel recorded neurons on the HD-MEA to better link the inferred connections to axonal/dendritic morphology and neuritic overlap. Such additional structural insights could also help to remove false positive connections.

### Conclusion

In sum, this study presents an analysis workflow to systematically assess algorithms that have been applied to the inference of neuronal connectivity from large-scale extracellular recordings. To this end, we utilized simulated ground-truth data and experimental HD-MEA/patch-clamp recordings, and compared statistically inferred connectivity across a range of different conditions in a standardized manner. Moreover, we introduced an *ensemble artificial neural network* (eANN), that can integrate the output of multiple inference algorithms, and probed whether this approach would lead to improved network-reconstruction performance. Our results demonstrate that the eANN can significantly improve inference performance and that the obtained network reconstruction represents more than just the sum of all methods.

## Methods and materials

Here, we describe the architecture and training scheme of the introduced *ensemble artificial neural network* (eANN). In S1 Text, we describe methodological details on the LIF simulations in (Sec A) and the model to infer synaptic connections from parallel recordings in (see Sec B). Next, we describe the HD-MEA and patch-clamp experiments, the *in vitro* culturing protocol and the performed data preprocessing steps (see Sec C). Details on the performed topological analyses are outlined in Sec D. Finally, the spike activity-based connectivity inference methods are described in Sec E.

### Ethics statement

All animal experiments were approved by the veterinary office of the Kanton Basel-Stadt (license #2358) and carried out according to Swiss federal laws on animal welfare.

### Architecture of the ensemble artificial neural network

The introduced *ensemble artificial neural network* (eANN) is a feed-forward network, that takes the weight *W* and the connectivity score matrices *S* of the six implemented connectivity methods (CI, sCCG, GLMCC, TE, dSTTC, and GLMPP) as input and provides as output the probabilities of whether the input belongs to an excitatory or an inhibitory connection, or whether the neurons are not connected at all (no connection). The eANN consists of two hidden layers with 10 units each and rectified linear units (ReLU) as non-linearity. The last layer is passed through a soft-max function to normalize the output. We trained the model on several leaky integrate-and-fire (LIF) neuron-based network simulations, which were subject to different inputs (see below). The trained network is the final eANN, which provides predictions based on the aggregated outputs of the other connectivity inference methods. The connectivity score of the eANN was calculated as *s*_*i*→*j*_ = max(*p*_E_, *p*_I_), where *p*_E_ and *p*_I_ are the eANN’s predicted likelihoods for an excitatory or inhibitory connection, respectively.

### Training the ensemble artificial neural network

We simulated 25 different LIF networks to generate training data for the eANN. To obtain the activity of distinct neuronal network dynamics, we used five different noise configurations *μ*_noise_, *σ*_noise_ (see Table A in S1 Text) and generated five networks with random connectivity for each configuration (connection probability: 0.05). Next, we simulated 1 h of LIF spiking activity as previously described (see Sec A1 in S1 Text). The experimental spiking input to the LIF network was always the same for the different training simulations. However, input activity from a different experimental recording was used for testing the eANN (i.e., the data reported in Fig 2). To train the network, we first obtained the connectivity output of all methods (CI, sCCG, GLMCC, TE, dSTTC, and GLMPP). Then, we took all synaptic connections, which make up 10% of the training set. For the remaining 90% of connections, we selected randomly unconnected pairs as negative examples. The connectivity score and weight provided by the traditional methods constituted the input variables. The training labels were set to 0 (no connection), 1 (excitatory connection), and 2 (inhibitory connection), and the eANN was trained by minimizing the cross-entropy loss on these data. To predict connections on a new dataset, we again first applied the original inference methods and then provided their aggregated result as input to the eANN. We noted that the compact network architecture prevented overfitting and ensured rapid convergence during training (see Fig I in S1 Text). Supplemental results for the eANN trained on simulated data that might recapitulate better the ongoing activity observed *in vivo* (e.g., showing fewer bursts), are provided in Fig L in S1 Text. The same LIF model was used, but with Poisson spiking as input, an 80:20 (E:I) neuron ratio, a connection probability of 0.02; an overview on all input parameters is provided in Table B in S1 Text.

## Supporting information

S1 Text Supporting information. (PDF)
