# Supplementary material for: Ensemble learning and ground-truth validation of synaptic connectivity inferred from spike trains
Source: PLoS Comput Biol. 2024 Apr 29;20(4):e1011964. doi: 10.1371/journal.pcbi.1011964 (PMC11081509; doi:10.1371/journal.pcbi.1011964)
Supplement: S1 Text — (PDF) [file pcbi.1011964.s001.pdf]

Supporting Information: Ensemble learning and ground-truth validation of synaptic connectivity inferred from spike trains

Christian Donner[1,*], Julian Bartram[2], Philipp Hornauer[2], Taehoon Kim[2], Damian Roqueiro[2], Andreas Hierlemann[2], Guillaume Obozinski[1], Manuel Schröter[2,*]

1 Swiss Data Science Center, ETH Zürich & EPFL, Zürich & Lausanne, Switzerland
2 Department of Biosystems Science and Engineering, ETH Zürich, Basel, Switzerland

Corresponding authors:
christian.research@mailbox.org
manuel.schroeter@bsse.ethz.ch

A In silico simulations of neuronal networks

A1 Leaky integrate-and-fire simulations

To test and compare network reconstruction performance across different algorithms, we adopted and modified the leaky integrate-and-fire (IF) network model approach proposed by Ren et al. [1]. The LIF simulations of the present study were performed with Brian2 [2]. The simulated network consisted of 300 neurons, balanced with 150 excitatory neurons and 150 inhibitory neurons. The differential equations of the IF model are

$$\begin{aligned} C_m \frac{\mathrm{d}V}{\mathrm{d}t} &= - g_{\mathrm{leak}}(V - V_{\mathrm{rest}}) - g_{\mathrm{AHP}}[\mathrm{Ca}^{2+}](V - V_{\mathrm{AHP}}) \\ &\quad + \mu_{\mathrm{noise}} + I_{\mathrm{noise}} + I_{\mathrm{syn}} \\ \frac{\mathrm{d}[\mathrm{Ca}^{2+}]}{\mathrm{d}t} &= - \frac{[\mathrm{Ca}^{2+}]}{\tau_{\mathrm{Ca}}} \\ \frac{\mathrm{d}I_{\mathrm{noise}}}{\mathrm{d}t} &= - \frac{I_{\mathrm{noise}}}{\tau_{\mathrm{noise}}} + \sigma_{\mathrm{noise}}\xi_t, \end{aligned}$$

where ξ_t describes a white noise process. While Ren et al. [1] have modeled the spontaneous I_{noise} as pink noise, here we approximate its dynamics as an Ornstein-Uhlenbeck process, which represents another common model for neuronal input [3, Chapter 8]. This approximation allowed simulating different dynamical regimes more systematically by changing the mean input μ_{noise} and the noise amplitude σ_{noise} (see Fig E **A**-**B**). The parameters describing the membrane potential dynamics were the membrane time constant $C_m = 500$ pF, the leak conductance 0.25 μS, and the resting potential $V_{\mathrm{rest}} = -65$ mV. For the after-hyperpolarizing (AHP) current, the conductance $g_{\mathrm{AHP}} = 0.15 \frac{\mathrm{S}}{\mathrm{m}^2}$, and the reversal potential $V_{\mathrm{AHP}} = -80$ mV. The dynamics of the $[\mathrm{Ca}^{2+}]$ were determined by the time-constant $\tau_{\mathrm{Ca}} = 100$ ms. When the membrane potential V surpassed the spiking

threshold $V_{\text{thresh}} = -50$ mV, a spike was registered, and the potential was reset to V_{rest}, and $[\text{Ca}^{2+}]$ was increased by 0.2 μM.

The synaptic input I_{syn} was composed of two sources: intrinsic spiking activity of the LIF network and experimental spiking activity. As external spiking activity, we used spike trains that were obtained from *in vitro* HD-MEA networks recording, which resembled the studied experimental conditions. As in the study by Ren et al. [1], the synaptic effect of spikes at time $\mathcal{T}_j = \{t^j_1, \dots, t^j_{N_j}\}$ neuron j connected to neuron i was modelled as

$$I^{j\to i}_{\text{syn}}(t) = w^{j\to i}_{\text{syn}} \sum_{t_s \in \mathcal{T}_j} H(t - t_s - \Delta t_{ij}) \frac{(t - t_s - \Delta t_{ij})}{\tau_{\text{syn}}} \exp\left(1 - \frac{(t - t_s - \Delta t_{ij})}{\tau_{\text{syn}}}\right),$$

where $H(\cdot)$ was the Heaviside function. The total synaptic input to a neuron i was then given by the sum of all synaptic currents in Eq. A1 the neuron was connected to. The connection probability among simulated LIF neurons was set to 5% and experimental input neurons were connected to LIF neurons with a probability of 10%. The synaptic weights were drawn randomly from a log-normal distribution $|w^{j\to i}_{\text{syn}}| \sim$ lognormal(-2.5,0.5) nA, and the weight was positive when j was an excitatory neuron and negative for inhibitory neurons. If the synaptic weight exceeded the interval [0.05 nA, 0.4 nA], it was set to the limits of this interval. Δt_{ij} was the synaptic time delay, which was given by distance_{ij}/conductionvelocity. The distance between neurons distance_{ij} was the Euclidean distance between the positions of neuron i and j. The positions for all neurons were drawn at random and uniformly from the rectangular area of the units from the experimental input recording (HD-MEA data). The conduction velocity for each neuron was drawn uniformly from the interval $[0.5\frac{\text{m}}{\text{s}}, 1\frac{\text{m}}{\text{s}}]$. Finally, $\tau_{\text{syn}} = 1$ms is the synaptic decay. For the *in silico* benchmarking analysis across different inference algorithms (see Fig 2), we used 1 h-long simulations. While we tried to match the statistics of the empirical spike-train data, future should attempt to infer the model parameter ranges directly from the respective data at hand [4].

A2 Simulations to validate connectivity inference from parallel HD-MEA/path-clamp recordings

The LIF framework described in the previous section was further adopted to also validate the methods applied to uncover synaptic connectivity from parallel HD-MEA/patch-clamp recordings (see Sec B and Fig E). For a detailed description of the HD-MEA/patch-clamp setup and the performed voltage-clamp (VC) measurements, we refer the reader to Sec C2, C4, and C5. To simulate the patch-clamp recordings at a control voltage $V_c = -55$mV, we constructed smaller subnetworks (LIF networks composed of n=10 neurons) with membrane currents of

$$I_m = -g_{\text{leak}}(V_c - V_{\text{rest}}) - g_{\text{AHP}}[\text{Ca}^{2+}](V_c - V_{\text{AHP}}) + \mu_{\text{noise}} + I_{\text{noise}} + I_{\text{syn}}.$$

These neurons got input similar to all other neurons in the network, but since they were in VC-clamp mode, they could not spike. Next, we simulated 10 min of these combined data, i.e., registering spike times of the 300 LIF neurons as described in the previous paragraph and the currents of 10 VC-clamped neurons. The analytical results obtained from this analysis are shown Fig E **C-D**.

B Inferring synaptic connectivity from parallel HD-MEA/patch-clamp recordings

Next, we describe the modeling approach to derive synaptic connectivity statistically from parallel HD-MEA/patch-clamp recordings. Our model was inspired by previous work [5], with some notable modifications, in order to have only one statistical test per potential connection.

B1 A regression analysis approach to estimate synaptic connectivity

In our model, we assumed that the recorded intracellular signal y_t is a linear superposition of two signals: i) a synaptic signal y^{syn} originating from synaptic signals from the extracellularly recorded presynaptic neurons, and ii), a residual signal ϵ, that accounts for all intrinsic, and extrinsic fluctuations, such as synaptic signals from neurons that could not be sampled. Hence, the full signal was given by

$$y_t = y_t^{\text{syn}} + \epsilon_t.$$

The modeled synaptic signal y_t^{syn} depends on the extracellularly recorded spiking activity of the presynaptic candidate neurons. Formally, the spike trains were a matrix with entries $s_{t,i} = 1$, if unit i elicited a spike at any time $[t\Delta, (t+1)\Delta)$; it was zero, if there was no spike. The synaptic signal y^{syn} was then modeled as a weighted sum of presynaptic signals $\boldsymbol{x}$

$$y_t^{\text{syn}} = \boldsymbol{\alpha}^\top \boldsymbol{x}_t^{\text{syn}},$$

with coupling parameters $\boldsymbol{\alpha} = (\alpha_1, \ldots, \alpha_N)^\top$. The presynaptic signals $\boldsymbol{x}^{\text{syn}}$ were modeled the spike trains convolved with a response kernel $k_n(\tau)$

$$x_{t,i}^{\text{syn}} = \sum_{\tau=1}^{L_k} k_{\tau,i} s_{t-\tau,i}.$$

The response kernel k had the parametric form of an alpha function, which has been previously used for modeling the form of postsynaptic potentials (PSPs) [6, Chapter 5]. It was given by

$$k_{\tau,i} = \begin{cases} \frac{(\tau\Delta-\delta_i)}{\tau_i} \exp\left(-\frac{\tau\Delta-\delta_i}{\tau_i}\right) & \text{if } \tau\Delta \geq \delta_i \\ 0 & \text{otherwise} \end{cases},$$

where τ_i the time constant, and δ_i the delay of the PSP. The latter is the main difference to Zhang et al. [5], which assumed a non-parametric footprint of the extracellularly recorded neurons on the intracellular signal. While the original model is likely more flexible, it requires multiple tests for each connection. In our model, however, we will subsequently only have one test per connection, i.e., whether the coupling parameter $\alpha_n \neq 0$. Everything, that could not be explained by the synaptic signal, such as transient fluctuations of the signal or the synaptic signals of unsampled neurons, we model by the following autoregressive process

$$\epsilon_t = h_0 + \sum_{\tau=1}^{L_h} h_\tau \epsilon_{t-\tau\Delta} + \xi_t = \hat{\epsilon}_t + \xi_t, \tag{S1}$$

where ξ_t was a Gaussian noise with standard deviation σ_y. Alternatively, ϵ_t can be written as $\epsilon_t = y_t - y_t^{\text{syn}}$. The former definition in Eq. (S1) only depends on the filter $\boldsymbol{h}$ and not on the couplings $\boldsymbol{\alpha}$. The alternative definition of ϵ_t is a function of $\boldsymbol{\alpha}$, but not of $\boldsymbol{h}$. This fact allowed us to define the alternating optimization scheme described in the following section.

B2 Fitting procedure

Given a recorded intracellular signal $y_{1:T}$ and extracellular spike trains $s_{1:T,1:N}$, we then sought to estimate the model parameters, i.e., the autoregressive filter $\boldsymbol{h}$, the synaptic coupling strengths $\boldsymbol{\alpha}$, the kernel parameters $\boldsymbol{\tau} = (\tau_1, \dots, \tau_N)^\top$, $\boldsymbol{\delta} = (\delta_1, \dots, \delta_N)^\top$, and finally, the noise parameters σ_y by the maximum likelihood principle. The previously described model defined the following (log) likelihood for the observed data

$$\begin{aligned} \ell(\boldsymbol{h}, \boldsymbol{\alpha}, \sigma_y, \boldsymbol{\tau}, \boldsymbol{\delta}; \lambda) &= \ln \prod_{t=1}^{T} p(y_i | \boldsymbol{y}_{0:t-1}, s_{1:t,1:N}, \boldsymbol{h}, \boldsymbol{\alpha}, \sigma_y, \boldsymbol{\tau}, \boldsymbol{\delta}) \\ &= -\frac{1}{2\sigma_y^2} \sum_{t=1}^{T} (y_t - (\hat{\epsilon}_t + y_i^{\text{syn}}))^2 - T \ln \sigma_y + \text{const.} \end{aligned} \tag{S2}$$

To avoid overfitting of the autoregressive filter $\boldsymbol{h}$, we included a regularizing term $\ell_{\text{reg}}(\boldsymbol{h})$ to penalize non-smooth filters. Formally, the regularization term is the second derivative of $\boldsymbol{h}$, i.e.,

$$\ell_{\text{reg}}(\boldsymbol{h}) = -\frac{1}{2} \|\Delta \boldsymbol{h}\|_2^2,$$

with Δ being the discrete Laplace operator. The optimal model parameters are given by

$$(\boldsymbol{h}, \boldsymbol{\alpha}, \sigma_y, \boldsymbol{\tau}, \boldsymbol{\delta}) = \text{argmin} \left[\frac{1}{2\sigma_y^2} \sum_{t=1}^{T} (y_t - (\hat{\epsilon}_t + y_t^{\text{syn}}))^2 + \lambda_h \ell_{\text{reg}}(\boldsymbol{h}) + \frac{T}{2} \ln \sigma_y^2 \right].$$

While there is no closed-form solution for this problem, we can derive analytic updates for the sub-problems by solving for $\boldsymbol{h}$, $\boldsymbol{\alpha}$, and σ_y separately. We, therefore, invoked an alternating optimization scheme. First, in order to find the optimal $\boldsymbol{h}$, we solved a linear problem. This problem is defined by computing the gradient of Eq. (S2) with respect to $\boldsymbol{h}$ and setting it equal to 0. In the same way, we then got the optimal couplings $\boldsymbol{\alpha}$. σ_y can be similarly derived analytically. What remained was to find the parameters $\boldsymbol{\tau}$ and $\boldsymbol{\delta}$, which were computed by gradient ascent maximization of Eq. (S2)). We then alternated the optimization procedure until the likelihood converged.

Finally, we sought to determine, which extracellular neurons (respectively their recorded spikes, $s_{1:T,i}$) were *de facto* connected to the neuron for which we had modeled the intracellular signal y. In other words, we asked which couplings were significantly non-zero (our null hypothesis was, that neurons are not connected, formally $H_0 : \alpha_i = 0$). To test for this hypothesis, we approximated the covariance of our estimate by the inverse Hessian matrix of the regularized log-likelihood (see Eq. (S2)) and used the absolute z-score $z_i = |\alpha_i|/\sigma_i$ as test-statistics. For the latter, σ_i^2 is the diagonal entry of the inverse Hessian matrix for couplings α_i. For

all couplings, where $z_i \geq \theta_\alpha$ the null hypothesis was rejected, i.e., these neurons were considered to be presynaptically connected to the patched neuron. The parameter θ_α was a threshold value that we derived by fitting the same model to jittered surrogate data (generated by adding Gaussian noise with a standard deviation of 5 ms as jitter to the extracellular detected spike times). We took the 95% quantile of the z-scores z_i, obtained by fitting the linear model to the jittered spike-train data, as a threshold.

C HD-MEA recordings

C1 High-density microelectrode arrays

To record from *in vitro* developing primary cortical networks, we used complementary-metal-oxide-semiconductor (CMOS) based high-density microelectrode arrays (HD-MEAs). These chips comprise 26,400 platinum microelectrodes (electrode size: 9.3 × 5.3 μm^2), with a 17.5 μm pitch and a total sensing area of 3.85 × 2.10 mm^2. HD-MEAs, as used in this study, allow for recordings from up to 1024 readout electrodes at the same time.

Two different types of HD-MEA systems, with comparable technical specifications, were used in this study: For the parallel HD-MEA/patch-clamp recordings, we used a custom single-well HD-MEA chip [7]. These HD-MEAs were bonded to printed circuit boards (PCBs), and a biocompatible epoxy (Epo-Tek 353ND, 35ND-T, Epoxy Technology Inc., USA) was used to encapsulate the bond wires and protect them from the medium. To decrease the impedance and to improve the signal-to-noise ratio (SNR), electrodes were coated with platinum black – deposited from a solution of hexachloroplatinic acid (7 mM, Sigma-Aldrich) and lead (2) acetate anhydrous (0.3 mM, Sigma-Aldrich) in distilled water, as described previously [8]. For the data and analysis presented in Fig 5, we used a commercially available 6-well HD-MEA plate by MaxWell Biosystems (Zurich, Switzerland). The extracellular signals were acquired at a sampling rate of 20 kHz, for the single HD-MEA, and at 10 kHz, for the 6-well HD-MEA plate. Before the cell plating, we sterilized HD-MEA chips for at least 30 min in 70% ethanol and washed them 3 × with sterile deionized water; the electrode array was then treated with 0.05% (v/v) poly(ethyleneimine) (Sigma-Aldrich) in borate buffer (Thermo Fisher Scientific, Waltham, Massachusetts, United States) at 8.5 pH for 40 min and then washed 3 × with sterile deionized water.

C2 Primary neuronal culture preparation

Rodent primary cortical neurons were prepared as previously described [8]: Cortices of embryonic day (E) 18/19 Wistar rats were dissociated in trypsin with 0.25 percent EDTA (Gibco), washed after 20 min of digestion in plating medium (see below), and finally gently triturated. Following cell counting with a hemocytometer, we seeded 15000-20000 cells (dataset 1: parallel HD-MEA/patch-clamp recordings; part of this data has been published in [9]), or 50,000 cells (dataset 2: HD-MEA network recordings; part of this data has been published in [10]) on each array, and placed it in a cell culture incubator for 30 min at 37°C/5% CO2. Then we added more plating medium carefully to each well (up to 1.5 mL). The plating medium was composed of 450 mL Neurobasal (Invitrogen, Carlsbad, CA, United States), 50 mL horse serum (HyClone, Thermo Fisher Scientific), 1.25 mL

Glutamax (Invitrogen), and 10 mL B-27 (Invitrogen). After two days, half of the plating medium was exchanged with the maintenance medium. For the maintenance medium, we added 50 mL Horse Serum (HyClone), 1.25 mL Glutamax (Invitrogen), and 5 mL sodium pyruvate (Invitrogen) to 450 mL of D-MEM (Invitrogen). The maintenance medium was exchanged twice a week and at least one day before the recording session. All animal experiments were approved by the veterinary office of the Kanton Basel-Stadt (license #2358) and carried out according to Swiss federal laws on animal welfare. For dataset 1 (parallel HD-MEA/patch-clamp recordings), the experiments were performed on days *in vitro* (DIV) DIV 17 (long-term HD-MEA baseline recording) and DIV 18 (parallel HD-MEA/patch-clamp measurements); for dataset 2 (1 h-long HD-MEA network recordings), datasets were recorded at DIV 14.

C3 High-density microelectrode array recordings

In order to select active recording sites on the HD-MEA for long-term network recordings, we first recorded the multi-unit activity for each electrode across the whole chip using a series of dense-block configurations. Activity during this pre-processing step ('activity scan') was assessed with an online sliding window threshold-crossing spike detection algorithm. The details for selecting the final recording electrodes are provided in the work by Bartram et al. [9] (dataset 1) and Akarca et al. [10] (dataset 2). Briefly, selecting a suitable network configuration involved a ranking of the online detected mean spike amplitudes (per channel) and that channels showed a minimum of spike activity. Each HD-MEA network configuration consisted of a max. of 1024 electrodes. The baseline recording for dataset 1 was composed of multiple network recordings on the day before the parallel HD-MEA/patch-clamp experiment (see Sec C3), yielding long network recordings of > 3 h duration. The network recording for the replication dataset (dataset 2) consisted of 1 h-long HD-MEA network recordings (n=6 cultures); here the network configuration was composed of up to 90 high-density electrode blocks (each block contained 4×4 electrodes).

C4 Spike-sorting of HD-MEA network recordings

HD-MEA network recordings were spike-sorted using a semi-automated processing pipeline. For dataset 1 (HD-MEA/patch-clamp recordings), we combined the baseline recordings with the data obtained during the patch-clamp session. For dataset 2, we used the 1 h-long network recording. To spike sort HD-MEA network recordings, we applied the publicly available software package Kilosort 2 (KS2) [11], using parameters adapted to our data. Following spike-sorting with KS2, we manually reviewed all neuronal units deemed 'good' using the general user interface (GUI) of phy2 (https://github.com/cortex-lab/phy). We excluded units that showed aberrant spike waveforms, and that did not meet some standard quality criteria (e.g., less than 5% refractory period violations), or that had too few spikes (> 1000 spikes for dataset 2).

C5 Patch-clamp electrophysiology

The parallel HD-MEA/patch-clamp experiments were performed using methodology introduced previously [12, 9]. To record from single neurons on the

HD-MEA, we transferred the chips to a custom patch-clamp rig and perfused them with warmed (32-34 °C) BrainPhys (BP). Patch-clamp recordings from cells located on the HD-MEA were obtained with borosilicate glass micropipettes (4-5 MΩ, Sutter Instruments, USA) containing (in mM): 85 caesium-gluconate, 60 CsCl, 10 Hepes, 4 Na_2ATP, 0.3 GTP, 2 $MgCl_2$, 0.1 EGTA, (pH 7.2-7.3; 280–290 mOsmol/l). Brief current-clamp recordings of spontaneous spiking were obtained, while synaptic activity was measured in voltage clamp mode at -70 mV holding potential. The high-chloride internal solution caused a shift in the GABA-A receptor reversal potential, which allowed us to record the synaptic activity of both GABAergic and glutamatergic synapses in one single recording. During the patch-clamp experiment, the same HD-MEA network configuration, as for the baseline recording, was used. This allowed to localize the patched cell on the HD-MEA, and to relate the intracellular obtained signals to the spike activity of the network. Patch-clamp recordings were carried out using an Axon Multiclamp 700B amplifier (Molecular Devices, USA), with digitization performed using an Axon Digidata 1440A (Axon Instruments). The recorded signals were low-pass filtered at 5 kHz and acquired with at least 20 kHz. Alexa 594 (20 μM, Thermo Fisher Scientific) was added to the internal solution to allow an assessment of the cell morphology. For details on the data, please see Bartram et al.[9].

D Topological characterization of networks

Here we provide more details on the procedures on how we inferred connectivity from HD-MEA derived spike trains, and how we analyzed this data using graph theoretical metrics (for details on the connectivity inference algorithms see Sec E, and Figs 2 and 5 in the main manuscript).

To ensure reliable connectivity inference from the spike-sorted HD-MEA network data, we applied several filtering steps. First, we restricted our analysis to randomly selected units (100) that had at least 1000 spikes over the course of the recording. Second, we only estimated connectivity for edges that had a spike-sorting index < 0.5, as suggested by Ren et al. [1], and that had at least 100 spikes in the pairwise CCG of units – in a time window relevant for fast synaptic interactions (+/- 20 ms). To generate jittered spike trains for units that passed the previously outlined quality criteria, and to be compatible with jittering code provided by [13], we only used uniquely occurring spike times, i.e., spikes that occurred at the zero-lag of the unit's auto-correlogram were removed. The aim of applying these arguably strict thresholds was to make sure that connectivity was estimated on a sufficient amount of data and to reduce the likelihood of false positive connections.

Next, we selected several common topological metrics and compared them across network inference methods (see results presented in Figs 2 and Fig 5). We categorize these metrics broadly into global and local topological features. The global features describe average statistics for the given networks, while the local metrics describe topological values resolved per individual node. The global metrics included the overall *network density*, the network *global efficiency*, the average *clustering coefficient*, the *modularity* [14] and the *small-world index* [15] of the network and the occurrence of *triplet-motifs* in the data [16]. All topological features were calculated using algorithms provided by the `Brain connectivity toolbox`[17]. We

briefly explain each metric below:

Degree. As the obtained binary graphs of the inferred networks were directed, the degree (k) denotes the sum of the in- and outgoing edges of the observed network.

Network density. The network density was defined as the number of significant edges divided by the number of all possible edges in the respective network.

Global efficiency. The global efficiency (E) is calculated as the average of the inverse shortest path length (L). It is a measure of the global integration of a graph [18].

Clustering coefficient. The clustering coefficient (C) measures the clustering of connections/nodes in the network. C for directed networks is calculated as the fraction of realized directed triangles around a node, i.e., the observed number of triangles divided by the number of all possible triangles [19].

Betweenness centrality. The betweenness centrality (B) was defined as the fraction of all shortest paths in the network that contain a given node. Nodes with high values of betweenness centrality therefore participate in many shortest paths [20].

Modularity index. The modularity index, Q, indicates how well a network can be partitioned into subgroups. We calculated it as proposed by [21].

Small-word index. The small-world index (S) of a binary network is usually defined by estimating two parameters: the characteristic path length of the network (L) and its average clustering coefficient (C). Both measures are normalized to appropriately randomized surrogate networks with the same number of nodes and edges [22]. Since some inferred networks contained disconnected nodes at the applied adaptive thresholds, and S is defined for connected networks, we used a variant of the small-world index [18]. We calculated S by dividing the normalized clustering coefficient ($C_{\text{norm.}}$) by the inverse of the normalized global efficiency ($1/E_{\text{norm.}}$).

Motifs. The frequency of triplet motifs was analyzed as proposed in previous work [16]. As for the small-world index, we generated appropriately randomized surrogate networks with the same number of nodes - and compared the empirical motif statistics to these randomized values. We compared a total of 13 different motifs.

To compare local topological features between the inferred networks and the LIF ground truth networks (see Fig 2), we computed Pearson's ρ between the set of values of the true network and the estimated network. For quantifying the difference between global connectivity features, we calculated the relative difference $(y_{\text{est}} - y_{\text{true}})/y_{\text{true}}$, where y_{est}, and y_{true} are the feature of the estimated and true network, respectively.

E Connectivity inference methods

In the following, we describe the connectivity inference algorithms implemented in this study. The considered algorithms include cross-correlogram (CCG)-based methods, methods based on information theory, neuronal synchrony, and finally, generalized linear model point processes. CCG-based methods have been widely applied to estimate synaptic connectivity from parallel recorded spike trains [23, 24]. Essentially, a CCG is a histogram of spike-time differences between two neurons i and j. We used the normalized CCG [25], defined as

$$\mathrm{CCG_{i\rightarrow j}}(\tau) = \sum_{\mathrm{t=1}}^{\mathrm{T}} \frac{(\mathrm{s_{t,i}} - \mu_{\mathrm{i}})(\mathrm{s_{t+\tau,j}} - \mu_{\mathrm{j}})}{\sigma_{\mathrm{i}}\sigma_{\mathrm{j}}},$$

where $s_{t,i}$ is the binary spike train of neuron i discretized in time bins with width Δ. $s_{t,i}$ is 1 if neuron i spiked between t and $t+\Delta$, and 0 otherwise. T is the number of time bins; μ_i and σ_i are the mean and standard deviation of the spike trains, respectively. To compute CCGs, we applied algorithms provided by the `Elephant` toolbox [26].

Coincidence index The first CCG-based method, implemented in this study, is termed *coincidence index* (CI, [27]). The CI was defined as

$$\mathrm{CI}_{i\rightarrow j} = \frac{\sum_{\tau=0}^{r} \mathrm{CCG_{i\rightarrow j}}(\tau)}{\sum_{\tau=0}^{T} \mathrm{CCG_{i\rightarrow j}}(\tau)}$$

where $r = T_{\mathrm{syn}}/\Delta$, and T_{syn} represents a time window in which synaptic effects are effective. Here we set $\Delta = 0.4$ms and $T_{\mathrm{syn}} = 6$ms. High CI values indicate an excess of spiking activity of neuron j after spikes of neuron i. As connectivity score, *connectivity score*, we took the absolute z-score $|\mathrm{CI}_{i\rightarrow j} - \mu_{i\rightarrow j}|/\sigma_{i\rightarrow j}$, where $\mu_{i\rightarrow j}$ and $\sigma_{i\rightarrow j}$ are the mean and standard deviation of CI values obtained from surrogate spike trains of the corresponding neuron pair (50 iterations). The surrogate spike trains were generated by jittering the spike times of neuron i with uniform noise $U(-1.5r\Delta, 1.5r\Delta)$. The jittering strongly decreased the pairwise correlations observed for interactions in the synaptic time window. As putative synaptic weight, W, we simply took the value $\mathrm{CI}_{i\rightarrow j}$. In the **spycon** toolbox (https://github.com/christiando/spycon), accompanying this study, we also provide the option to apply a deconvolution step for all CCG-based methods, as proposed by [28]. While we did not see performance increases for our data, such preprocessing might become relevant for spike-train data with strong autocorrelations.

Smoothed CCG While the CI relies on the generation of jittered surrogate spike-train data, Stark et al. [29] proposed a simple smoothing procedure in combination with a statistical test to assess whether the CCG deviates from the H_0 hypothesis, i.e., that two neurons are synaptically not connected. Using this approach, the CCG is convolved with a Gaussian kernel, which is considered to have a similar effect on the CCG as obtaining a threshold value through spike-train jittering. Avoiding the jittering operation makes this approach computationally more efficient. We took the negative logarithm of the p-value as connectivity score, and as weights W the synaptic strength, as described previously [30]. Throughout the

manuscript, we referred to this algorithm as the *smoothed CGG* (sCCG) method.

Generalized linear model CCG An alternative method, combining the CCG approach with generalized linear models (GLMs), was proposed by Kobayashi et al.[31]. This approach decomposes CCGs into a slow and a fast fluctuating component. The model assumes that slow fluctuations, as observed in a pairwise CCG, can be regarded as the background activity within the network. Only fast short-latency fluctuations, that is, prominent peaks and troughs in the pairwise CCG that exceed the background activity, and that happen within the synaptic time window, should be considered as putative excitatory and inhibitory connections. Hence, Kobayashi et al.[31] proposed a parametric model, namely a GLM, to fit empirical CCGs. Formally, the GLM was given by

$$c(t) = \exp\left[a(t) + J_{ij}f(t) + J_{ji}f(-t)\right],$$

where $c(t)$ is the co-firing rate for a timeshift bin t. $a(t)$ models the slow fluctuation that encodes the background activity. $f(t)$ models the synaptic interaction, which is given in the form of a decaying exponential function $f(t) = \exp(-\frac{t-d}{\tau})$ (for $t > d$, $f(t) = 0$ otherwise), where d is the synaptic delay and τ is the time constant of the decay. The parameters J_{ij} and J_{ji} are the coupling strengths from neuron i to j and j to i, respectively. Given a CCG from observed data, the maximum a posterior (MAP) estimate for parameters $\theta = \{J_{ij}, J_{ji}, a(t)\}$ is then obtained by numerical optimization. For details, the reader is referred to the original publication [31]. A similar approach has also been suggested by Ren et al. [1]. As *connectivity score*, we take the z-score derived in [31, Eq. 14] given by $s_{i\to j} = |J_{ij}|\sqrt{\tau c(0)}/1.57$. For the synaptic weight, Kobayashi et al.[31] proposed to transform the parameter J_{ij} heuristically to the size of a post-synaptic potential by the following formula $w_{i\to j} = J_{ij}/a$, where the factor $a = 0.39$, if there is a putative excitatory connection, i.e., $J_{ij} > 0$. On the other hand, for inhibitory connections, i.e., $J_{ij} < 0$, they used $a = 1.57$.

Transfer entropy Another important class of methods that has been widely applied to probe neuronal interactions, and to reconstruct neuronal networks, relies on information theory. The present study focussed on *transfer entropy* (TE) [32] and uses algorithms by the *IDTxl* toolbox [33] to estimate the functional connectivity between neurons. TE quantifies the "amount of predictive information" [34], between two processes – respectively, here, the spike trains of a source neuron i and a target neuron j. In brief, TE measures if including information on the spiking activity of neuron i, adds to the prediction of the future activity of neuron j, which goes beyond the information that is contained in the past activity of j alone. In the present study, we computed the TE on discretized spike train data with bins of size Delta=5 ms. As for the CI method, we used the absolute z-score $s_{i\to j} = |\mathrm{TE}_{i\to j} - \mu_{i\to j}|/\sigma_{i\to j}$ as connectivity score. Again, $\mu_{i\to j}$, $\sigma_{i\to j}$ are the mean and standard deviation of the TE values computed from jittered spike trains (50 iterations). The jitter noise was uniform$(-3.5\Delta, 3.5\Delta)$. As the weight of a connection, we considered the value $\mathrm{TE}_{i\to j}$. Compared to the other implemented methods, the TE value is unsigned, i.e., it is always positive and, in that regard, could not distinguish between excitatory and inhibitory connections.

There are considerable limitations associated with the use of TE as a direct measure of coupling strength, as it may be confounded by the firing rate of neurons, the dynamical state of the network, the used embedding dimensions, and several other factors [34]. Future studies should implement and probe more recent TE variants that have been specifically developed for spike-train data [35] and that have addressed some of the limitations of current TE algorithms.

Directed spike tiling coefficient Cutts et al.[36] introduced the *spike time tiling coefficient* (STTC) to quantify synchronicity between spike trains. This method is computationally fast and has recently gained a lot of popularity. While the original method provided a measure of undirected pairwise correlation or functional connectivity, we modified the approach by Cutts et al. to a directed variant, which we call the *directed STTC* (dSTTC). In the following, we outline the dSTTC between the spike trains of neuron i and j. As in the original method, we defined a synaptic time window $\Delta_{\text{syn}} = 7\text{ms}$. T_i^{pre} is the proportion of the total recording time, which is covered by time windows Δ before the spikes of neuron i. We note, that the times of overlapping windows are just considered once. Similarly, we define T_j^{post} for the proportion of recording time, which is covered by windows Δ_{syn} following spikes of neuron j. Furthermore, we define $P_{j\to i}^{\text{pre}}$ as the proportion of spikes of neuron j, that lies in the time windows Δ_{syn} preceding the spikes of neuron i. Similarly, $P_{j\to i}^{\text{post}}$ is the proportion of spikes of neuron i following the spikes of neuron j. Finally, we defined the dSTTC as

$$\text{dSTTC}_{j\to i} = \frac{1}{2}\left(\frac{P_{j\to i}^{\text{pre}} - T_i^{\text{pre}}}{1 - P_{j\to i}^{\text{pre}} T_i^{\text{pre}}} + \frac{P_{j\to i}^{\text{post}} - T_j^{\text{post}}}{1 - P_{j\to i}^{\text{post}} T_j^{\text{post}}}\right),$$

which can result in values in the range $[-1, 1]$, as for the undirected original implementation by Cutts et al.[36]. The intuition behind the presented statistic is, that an excess of spiking of neuron i, that follows the spiking of neuron j, should indicate an excitatory connection. In this case, the dSTTC attains positive values. On the contrary, for inhibitory connections, we would expect a scarcity, or reduction, of spiking instead. In the latter case, the dSTTC would then result in more negative values. If the spikes of neurons i and j are occurring randomly, the dSTTC is expected to be close to 0. It should be noted, however, that these statements are based on the assumption, that the recorded data is stationary, i.e., there are no gross fluctuations in the firing rates. In the experiments at hand, however, this is rarely the case, due to transients in the firing rate and/or network burst dynamics. To mitigate such effects due to violations of the stationarity assumption, we resorted again to absolute z-score values for *connectivity score* and calculate $s_{j\to i} = \frac{|\text{dSTTC}_{j\to i} - \mu_{j\to i}|}{\sigma_{j\to i}}$, where $\mu_{j\to i}$ and $\sigma_{j\to i}$ are the mean and standard deviation of the dSTTC values obtained from jittered spike trains. As in Cutts et al. [36], we used jitter noise $\text{Uniform}(-3.5\Delta_{\text{syn}}, 3.5\Delta_{\text{syn}})$. As weight $w_{j\to j}$ of a putative connection between two neurons, we took the raw $\text{dSTTC}_{j\to i}$ value.

Point process generalized linear model All presented algorithms so far were pairwise connectivity-inference methods. That is, they considered only two neurons at a time and neglected the potentially contributing effect of the activity of other neurons in their calculation. The *Generalized Linear Model Point Processes* (GLMPP) approach, however, is a framework that

does consider such network interrelation – and has been previously used to probe connectivity [37]. This approach models the spiking of neuron i by a point process with rate $\lambda_i(t|\mathcal{H}_t)$, where $\mathcal{H}_t$ is the recorded spiking history up to time t. Here, we will assume that the rate model is

$$\lambda_i(t|\mathcal{H}_t) = f_i\left(\theta_i + \sum_{j=1}^{N} J_{j\to i}\phi_j(t)\right), \tag{S3}$$

where the feature $\phi_j(t)$ is the spike train of neuron j convolved with a causal exponential function with decay $\tau = 5$ms. f is a monotonically increasing non-negative function. In this model, the parameters of interest are the coupling $J_{j\to i}$ for $i \neq j$; note that$J_{i\to i}$ models how the neuron's activity influences itself, such as, for example, the refractory period following a spike. For an excitatory connection $i \to j$, we expected, that the rate $\lambda_i(t|\mathcal{H}_t)$ increases after spikes of neurons j, and hence $J_{j\to i}$ should be positive. The contrary holds for inhibitory connections. If $J_{j\to i}$ was close to 0, this should indicate that no connection is present. We assumed a Gaussian prior distribution $J_{j\to i} \sim \mathcal{N}(0, \sigma_J^2)$ and $\theta_i \sim \mathcal{N}(\mu_\theta, \sigma_\theta^2)$. We intended to obtain the posterior distribution of the model parameters given the recorded spike trains. In general, this is not straightforward for a model defined by Eq. (S3). However, by choosing the $f(\cdot)$ to be a scaled sigmoid as in [38, 39], efficient variational algorithms have been developed to obtain an approximate Gaussian posterior distribution [40, 41] over the parameters θ_i, and $J_{j\to i}$ via variational inference. Hence, once we have the approximate posterior density over parameters θ, J, we define the connectivity score for a given connection as $s_{j\to i} = |\mu_{j\to i}|/\sigma_{j\to i}$, where $\mu_{j\to i}, \sigma_{j\to i}$ are the mean and standard deviation of the posterior estimate for $J_{j\to i}$. As connection weight, we used the coupling value $J_{j\to i}$.

Model specification and inference Given the spike trains of several neurons, we can readily compute the features at any time

$$\phi_j(t) = \sum_{t_j \in \text{spikes of } j} \int k(\tau)\delta(t-\tau-t_j)\mathrm{d}\tau$$

where $k(\tau) = \frac{1}{\tau_k}\exp(-\frac{\tau}{\tau_k})$ and $\tau_k = 5$ms. In the following, we used the methodology of [40, 41] to fit the point process. In order to do so, we needed to define the non-linearity f_i in rate in Eq. S3 as scaled sigmoid

$$\lambda_i(t) = \bar{\lambda}_i\sigma(\mathbf{J}_i^\top \boldsymbol{\theta}(t)),$$

where $\bar{\lambda}_i > 0$, $\mathbf{J}_i^\top = (\theta_i, J_{1\to i}, \cdots, J_{N\to i})^\top$ and $\boldsymbol{\theta}(t) = (1, \phi_1(t), \cdots, \phi_N(t))^\top$. For notational convenience, we dropped the conditioning on the history $\mathcal{H}_t$. The likelihood of a point process [42] for spikes of neuron i $\mathcal{T}_i = \{t_1^i, \dots, t_{N_i}^i\}$ is

$$p(\mathcal{T}_i|\mathbf{J}_i) = \prod_{t_i\in\mathcal{T}_i} \lambda_i(t)\exp\left(-\int_0^T \lambda_i(t)\mathrm{d}t\right).$$

We assumed a Gaussian prior over the parameters $\mathbf{J}_i$ and a Gamma distribution prior over $\bar{\lambda}_i$. With this setting, we can utilize the augmentation scheme and the variational approach described in [41] to obtain an approximate Gaussian posterior over the $\mathbf{J}_i$, which we then used for the final connectivity.

F Statistical thresholding of connectivity and evaluation of reconstruction performance

Threshold selection After inferring connectivity from either simulated or experimentally obtained spike-train data, several downstream analyses of this work required binary graphs. Hence, the connectivity matrices had to be thresholded. Selecting an appropriate threshold is a delicate task since it can affect the interpretation of the graph structure and its organizational properties. In the present study, we applied three different strategies. For the comparison of different inference methods on the LIF network data (see Fig 2), we performed a search for the threshold that yielded the maximal Matthews correlation coefficient (MCC, see Sec. F, i.e., the highest similarity to the underlying ground-truth graph. Such an approach has been previously applied in the literature [31], and does allow for a fair performance comparison across inference methods. However, since such a threshold optimization is not applicable to experimental data, we also report results using a second approach, that relied on a global adaptive thresholding logic (see Figs 5 and B). We, therefore, recalculated connectivity on jittered surrogate data (Gaussian jitter with a standard deviation of 10ms), and defined an absolute global threshold as the $(1-\alpha)*100\%$ quantile of the resulting connectivity score distribution. Here α can be interpreted as an expected false positive rate. The aim of this procedure was to destroy all short-latency synchronization by temporal jittering while keeping the firing rate dynamics intact. The jittered distribution of the connectivity score values then reflected the null hypothesis, i.e., that there were no connections. For the HD-MEA recordings, we varied α-values from 0.05 to 0.001 (see Fig 5). The data was only jittered once, which made this approach computationally fast. Finally, to show that topological results were stable across the selected statistical thresholds, we also applied proportional thresholds (see D Fig). With these thresholds, we probed graph metrics at a specific network density (e.g., 5%) and compared the topological properties of networks at a defined percentage of the strongest connections.

Performance measures To quantify the network-reconstruction performance across all inference algorithms, we applied standard validation measures, commonly used for classification tasks. The first metric is the *average precision score* (APS), which is threshold-free, i.e., there is no need to specify a threshold. The APS is calculated from the area under the *precision-recall* curve and is formally defined as $\mathrm{APS} = \sum_n (R_n - R_{n-1}) P_n$. R_n, P_n are recall and precision if the nth smallest connectivity score would be selected as threshold. The APS provides values between 1 (perfect classification possible) and 0 (no connection is correctly classified without misclassifying all unconnected pairs). As a second performance measure, we implemented the *Matthews correlation coefficient* (MCC). The MCC requires a binary connectivity matrix, respectively matrices, to compare networks. It is defined as

$$\mathrm{MCC} = \frac{n_{\mathrm{TP}} n_{\mathrm{TN}} - n_{\mathrm{FP}} n_{\mathrm{FN}}}{\sqrt{n_{\mathrm{P}} n_{\mathrm{N}} n_{\mathrm{PP}} n_{\mathrm{PN}}}},$$

where $n_{\mathrm{TP}}, n_{\mathrm{TN}}$ are the number of true positives and false negatives, respectively, i.e., it is a measure of whether the algorithm classified the putative connections correctly. $n_{\mathrm{FP}}, n_{\mathrm{FN}}$ denote the numbers of false positives and

false negatives. $n_{\mathrm{P}}, n_{\mathrm{N}}$ are the total number of connections and unconnected pairs of the ground-truth data. $n_{\mathrm{PP}}, n_{\mathrm{PN}}$ are the number of predicted positives (connection) and predicted negatives (non-connections), respectively. The MCC gives values between 1, for perfect classification, and -1 for the worst outcome.

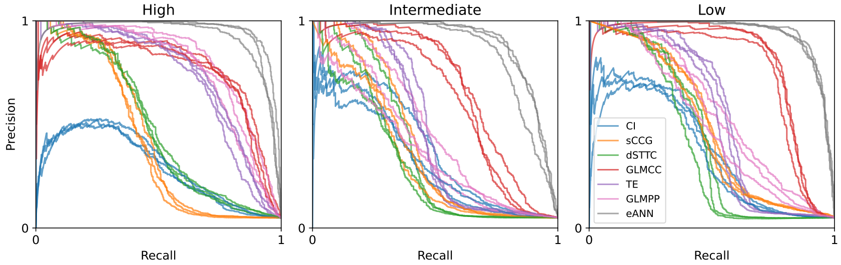

Fig A: **Precision-recall curves for connectivity inference performed on simulated data.** Precision-recall curves for all algorithms; analysis was performed on the same data as in Fig 2; three lines per method for three networks ($N = 100$) that were fitted for the high, intermediate, and low-burst condition. The more these curves extend to the upper right corner, the better classifications can be achieved. The eANN is the most robust method across all three conditions. The APS values in Fig 2**D** correspond to the area under these curves.

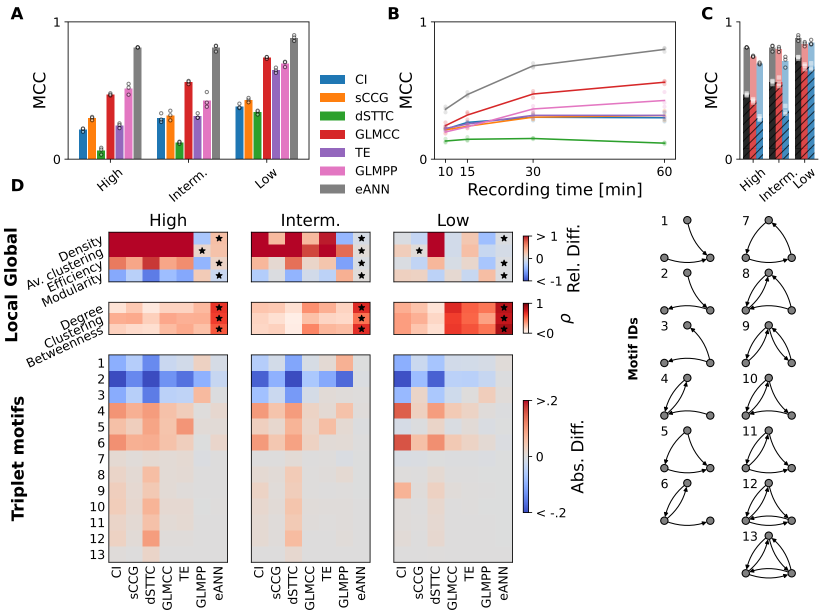

Fig B: **Network-reconstruction performance with a global adaptive threshold.** Results corresponding to Fig 2 achieved with an adaptive threshold selection. **A** The MCC of different connectivity methods. Dots depict the performance obtained from fits on three different subnetworks of the same simulation. **B** Classification performance (MCC) as a function of recording time. **C** MCC for each type of connectivity, that is, excitatory (E, in red), inhibitory (I, in blue), combined (E+I, in black). Correspondingly, the performance gains achieved by the eANN are plotted in shades of red, blue, and black. **D** Quality of topological feature reconstruction for the inferred network across the three dynamical regimes. In the upper panel, the relative difference between four global features (network density, av. clustering, and efficiency) is shown. Panels in the middle indicate the Pearson correlation coefficient for local features (per node of the network) between the true and the inferred network. Black stars indicate which method performed best. In the lower panels, we show the absolute difference of triplet-motif frequencies between ground truth and the different estimated networks.

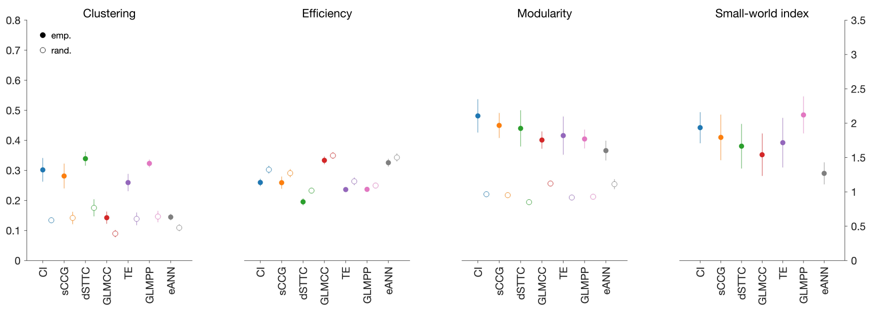

Fig C: **Comparison of in vitro neuronal network topology across inference methods.** The panel shows four topological metrics (average clustering, efficiency, modularity, and small-world index) inferred from HD-MEA network recordings of *in vitro* developing neuronal networks at DIV 14. Connectivity analysis is performed on graphs containing the strongest 5% of connections (proportional thresholding). Each panel depicts one topological measure; the colors correspond to the seven inference algorithms; colored circles correspond to the values obtained from the empirical data; the white-filled circles correspond to the surrogate networks (randomly rewired networks). In contrast to the analysis in Fig 5**H**, this figure depicts all networks with the same number of edges.

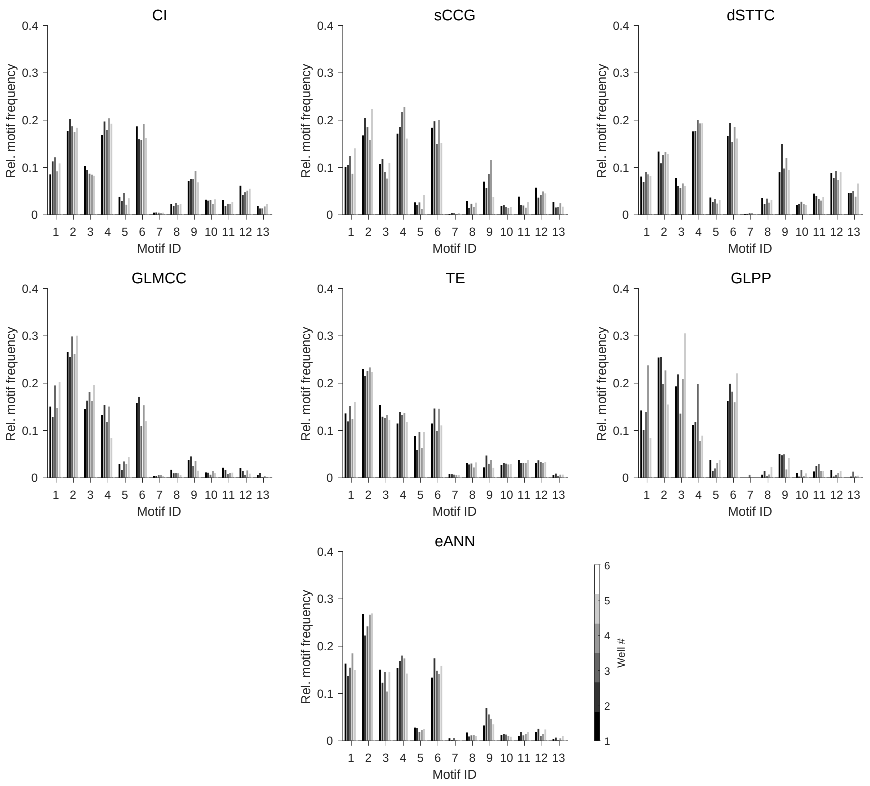

Fig D: **Triplet-motif frequency across inference methods and in vitro neuronal networks.** The triple-motif frequency was very similar within each method class but differed considerably between some inference methods (see Fig 5**I** and 5**J**). Depicted motif frequencies were normalized, for each culture, by the total amount of observed motifs (binarization threshold: α=0.01; network size: 100 units/network; recording duration: 1 h; age: DIV 14).

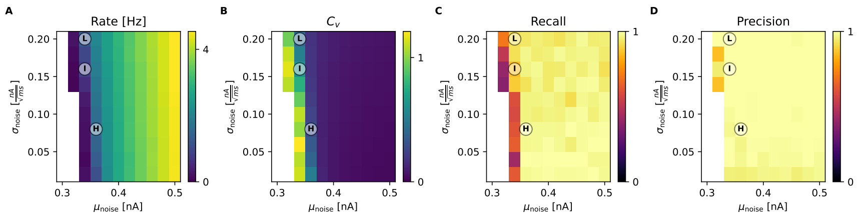

Fig E: **In silico validation of synaptic connectivity inference from simultaneous extra- and intracellular recordings. A-B** An overview of the modeled dynamic range to validate the PSC connectivity inference method. The average firing rate and Fano factor are shown for different simulations. Simulations with a rate < 0.5 Hz were excluded. Circles correspond to simulations with **L**ow, **I**ntermediate, and **H**igh burst rate in Fig 2. **D-E** Display of recall and precision values for the in silico validation of the patch-clamp connectivity inference method. Performance was averaged across in silico VC recordings from 10 different neurons.

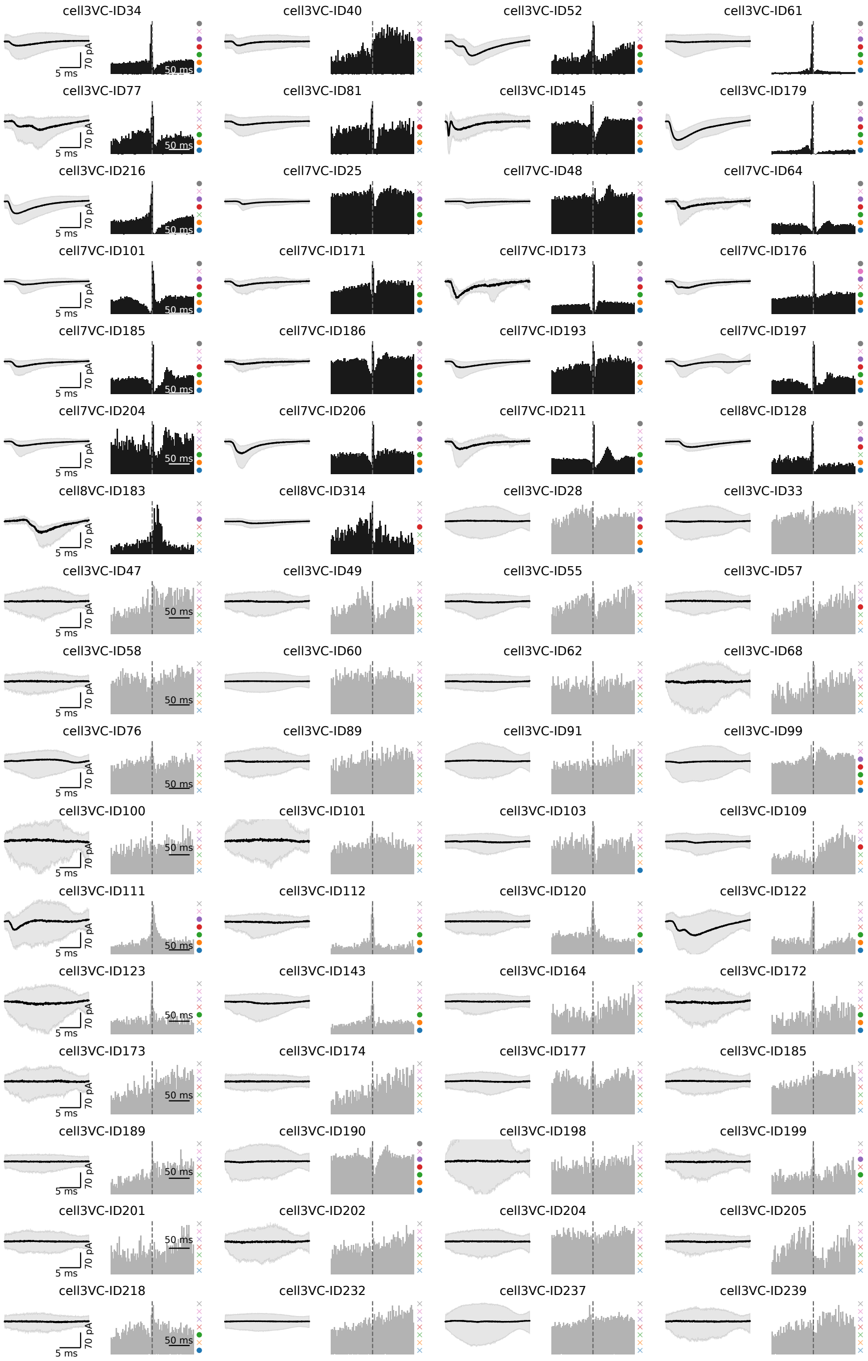

Fig F: **Estimating synaptic connectivity in the parallel HD-MEA/patch-clamp dataset** Here, we show all 26 experimentally obtained connections inferred by the parallel HD-MEA/patch-clamp recordings, together with all pairs for which no connection was found. We show the PSCs, the corresponding spike-train CCG, and the classification of the different connectivity methods similar to Fig 4.

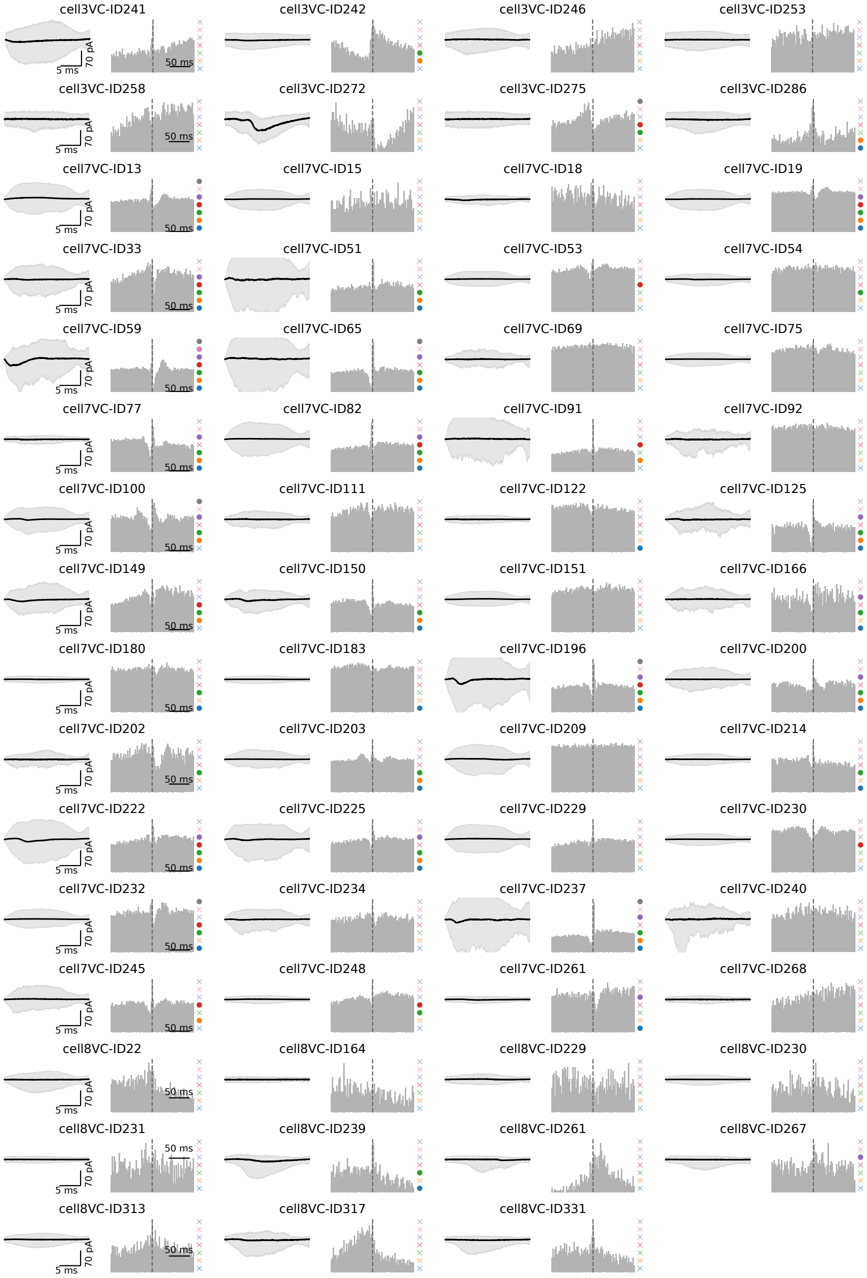

Fig F: **Estimating synaptic connectivity in the parallel HD-MEA/patch-clamp dataset (continued).**

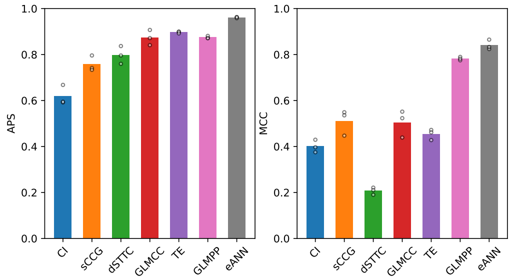

Fig G: **Comparing connectivity-inference performance with varying excitatory/inhibitory neuron ratios (in silico)** Network-reconstruction performance for all inference methods on simulated data with an 80/20 excitatory/inhibitory neuron ratio. The average spiking rate was 1.65 Hz and the burst rate was 0.33 Hz, which is closest to the intermediate burst regime in Fig 2.

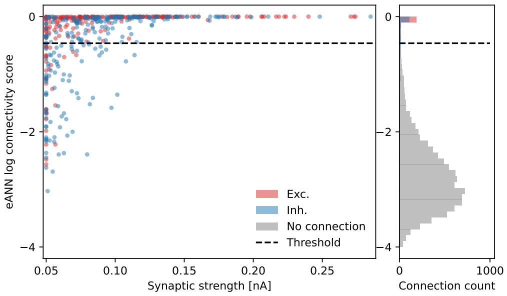

Fig H: **Relation of eANN connectivity score and synaptic strength values (in silico).** The panel on the left depicts the synaptic strength values of the simulated data (on the x-axis) against the log-transformed connectivity score values inferred by the eANN (on the y-axis). Results indicate that lower eANN connectivity scores are more likely for weaker synaptic connections. The right panel shows a histogram of the (log) connectivity score values. The no-connections are omitted in the left panel because their synaptic strength is not defined. The plot is based on the data generated for the intermediate-burst regime shown in Fig 2.

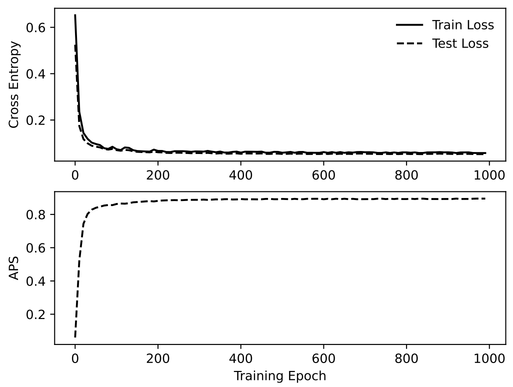

Fig I: **Training and test loss during eANN training.** The top panel depicts the cross-entropy loss for the training set and a test set (intermediate-burst regime). The lower panel depicts the APS for the test set during eANN training.

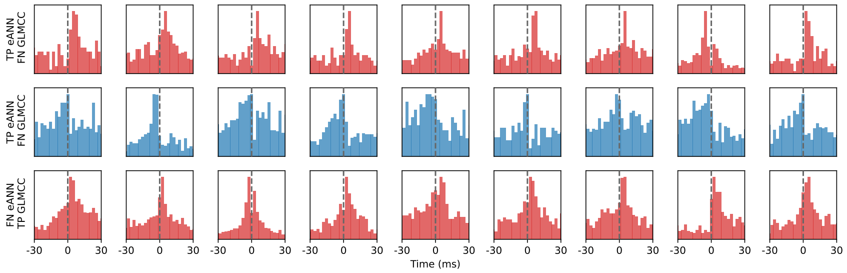

Fig J: **Comparison of eANN and GLMCC inference on in silico data.** Example cross-correlograms to compare connectivity inference of the eANN and the GLMCC method on selected pairs (TP = true positive, NF = false negative). The color of the bars indicates if the ground truth connection was excitatory (red) or inhibitor (blue).

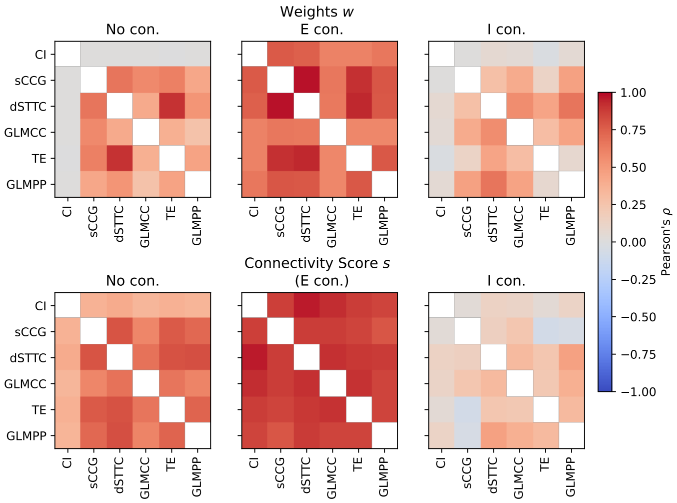

Fig K: **Correlation of connectivity weights and connectivity scores for eANN input data.** The first row shows all-to-all Pearson correlation matrices for the connectivity weight values, calculated across all inference methods; the values are depicted for un-connected pairs ('No con.'; on the left), excitatory connections ('E con.', in the middle), and inhibitory connection ('I con.', on the right). Correspondingly, the second row shows all-to-all Pearson correlation matrices for the connectivity score. The analysis was performed for data during the intermediate-burst regime (Fig 2).

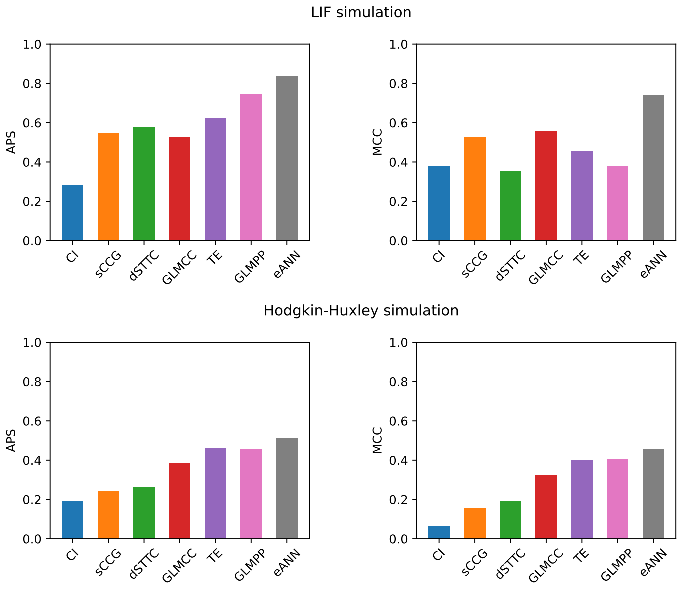

Fig L: **Benchmarking methods on ongoing activity of LIF and Hodgkin-Huxley simulations.** To test the eANN on simulated data that resembles more the ongoing activity in vivo, we probed network reconstruction performance of a subnetwork simulation comprising 100 (80/20 E/I) LIF (top) and Hodgkin-Huxley (HH) neurons; the HH data were adopted from [43] (bottom). For these data, the eANN was retrained on LIF simulations A with different firing rates. To this end, we time-shuffled the experimental input spiking data for the LIF network and used the parameters described in Table B to generate the training data. With these data, the eANN was trained exactly as before, and tested on the LIF and Hodgkin-Huxley data.

μ_{noise} [nA]	σ_{noise} [$\frac{\text{nA}}{\sqrt{\text{ms}}}$]	Spike rate [$\frac{\text{spikes}}{\text{s}\cdot\text{neuron}}$]	C_v	Burst rate [$\frac{\text{Bursts}}{\text{s}}$]
0.34	0.2	1.4	0.8	0.1
0.34	0.16	1.2	1.1	0.1
0.36	0.08	1.7	0.6	0.8
0.42	0.08	3.2	0.2	5.6
0.40	0.18	2.8	0.3	3.6

Table A: **Training simulations for the eANN.** This table provides an overview of the parameters ($\mu_{\text{noise}}, \sigma_{\text{noise}}$) and resulting spiking statistics (spike rate, coefficient of variation, and burst rate) for the LIF simulations (Sec A) from which the eANN training set was constructed.

μ_{noise} [nA]	σ_{noise} [$\frac{\text{nA}}{\sqrt{\text{ms}}}$]	Spike rate [$\frac{\text{spikes}}{\text{s}\cdot\text{neuron}}$]	C_v
0.36	0.2	11.7	0.3
0.34	0.2	3.0	0.8
0.35	0.2	7.3	0.5
0.33	0.3	4.3	0.7
0.30	0.5	3.9	0.8
0.35	0.10	1.5	0.9

Table B: **Training and test simulations for the eANN with simulated ongoing activity data.** This table provides an overview of the parameters ($\mu_{\text{noise}}, \sigma_{\text{noise}}$) and resulting spiking statistics (spike rate, coefficient of variation) for the LIF simulations used for the eANN training (first 5 rows) and testing (last row) in Fig L.

References

1. Ren N, Ito S, Hafizi H, Beggs JM, Stevenson IH. Model-based detection of putative synaptic connections from spike recordings with latency and type constraints. Journal of Neurophysiology. 2020;124(6):1588–1604. doi:10.1152/JN.00066.2020.

2. Stimberg M, Brette R, Goodman DFM. Brian 2, an intuitive and efficient neural simulator. eLife. 2019;8. doi:10.7554/ELIFE.47314.

3. Gerstner W, Kistler WM, Naud R, Paninski L. Neuronal dynamics: From single neurons to networks and models of cognition. 2014;.

4. Lueckmann JM, Goncalves PJ, Bassetto G, Öcal K, Nonnenmacher M, Macke JH. Flexible statistical inference for mechanistic models of neural dynamics. Advances in Neural Information Processing Systems. 2017;30.

5. Zhang Y, Xiao Y, Zhou D, Cai D. Spike-triggered regression for synaptic connectivity reconstruction in neuronal networks. Frontiers in Computational Neuroscience. 2017;11:101. doi:10.3389/fncom.2017.00101.

6. Dayan P, Abbott L. Theoretical neuroscience: computational and mathematical modeling of neural systems; 2005. Available from: `https://books.google.de/books?hl=en&lr=&id=fLT4DwAAQBAJ&oi=fnd&pg=PR13&dq=theoretical+neuroscience&ots=-WkoyZ72Sx&sig=RZD0iNFWInJ8mktfYGGFvLB-yaw`.

7. Müller J, Ballini M, Livi P, Chen Y, Radivojevic M, Shadmani A, et al. High-resolution CMOS MEA platform to study neurons at subcellular, cellular, and network levels. Lab on a Chip. 2015;15(13):2767–2780. doi:10.1039/C5LC00133A.

8. Bakkum D, Frey U, Radivojevic M, … TRN, 2013 u. Tracking axonal action potential propagation on a high-density microelectrode array across hundreds of sites. naturecom. 2013;doi:10.1038/ncomms3181.

9. Bartram J, Franke F, Kumar SS, Buccino AP, Xue X, Gänswein T, et al. Parallel reconstruction of the excitatory and inhibitory inputs received by single neurons reveals the synaptic basis of recurrent spiking. bioRxiv. 2023; p. 2023.01.06.523018. doi:10.1101/2023.01.06.523018.

10. Akarca D, Dunn AWE, Hornauer PJ, Ronchi S, Fiscella M, Wang C, et al. Homophilic wiring principles underpin neuronal network topology in vitro. bioRxiv. 2022; p. 2022.03.09.483605. doi:10.1101/2022.03.09.483605.

11. Pachitariu M, Steinmetz NA, Kadir SN, Carandini M, Harris KD. Fast and accurate spike sorting of high-channel count probes with KiloSort. Advances in Neural Information Processing Systems. 2016;29.

12. Jäckel D, Bakkum DJ, Russell TL, Müller J, Radivojevic M, Frey U, et al. Combination of High-density Microelectrode Array and Patch Clamp Recordings to Enable Studies of Multisynaptic Integration. Scientific Reports 2017 7:1. 2017;7(1):1–17. doi:10.1038/s41598-017-00981-4.

13. Marshall N, Timme NM, Bennett N, Ripp M, Lautzenhiser E, Beggs JM. Analysis of Power Laws, Shape Collapses, and Neural Complexity: New Techniques and MATLAB Support via the NCC Toolbox. Frontiers in Physiology. 2016;7:191703. doi:10.3389/FPHYS.2016.00250/BIBTEX.

14. Newman MEJ. Spectral methods for network community detection and graph partitioning. Physical Review E - Statistical, Nonlinear, and Soft Matter Physics. 2013;88(4). doi:10.1103/PhysRevE.88.042822.

15. Watts DJ, Strogatz SH. Collective dynamics of 'small-world' networks. Nature 1998 393:6684. 1998;393(6684):440–442. doi:10.1038/30918.

16. Sporns O, Kötter R. Motifs in Brain Networks. PLoS Biology. 2004;2(11). doi:10.1371/JOURNAL.PBIO.0020369.

17. Rubinov M, Sporns O. Complex network measures of brain connectivity: Uses and interpretations. NeuroImage. 2010;52(3):1059–1069. doi:10.1016/J.NEUROIMAGE.2009.10.003.

18. Latora V, Marchiori M. Efficient Behavior of Small-World Networks. Physical Review Letters. 2001;87(19):198701. doi:10.1103/PhysRevLett.87.198701.

19. Fagiolo G. Clustering in complex directed networks. Physical Review E - Statistical, Nonlinear, and Soft Matter Physics. 2007;76(2):026107. doi:10.1103/PHYSREVE.76.026107/FIGURES/9/MEDIUM.

20. Kintali S. Betweenness Centrality : Algorithms and Lower Bounds. 2008;.

21. Newman MEJ. Fast algorithm for detecting community structure in networks. Physical Review E - Statistical Physics, Plasmas, Fluids, and Related Interdisciplinary Topics. 2004;69(6):5. doi:10.1103/PHYSREVE.69.066133.

22. Maslov S, Sneppen K. Specificity and stability in topology of protein networks. Science. 2002;296(5569):910–913. doi:10.1126/SCIENCE.1065103/ASSET/C5A93A84-FAD7-4B06-A02C-1ABDC4984326/ASSETS/GRAPHIC/SE1720437003.JPEG.

23. Aertsen A, Gerstein GL, Habib MK, Palm G. Dynamics of neuronal firing correlation: modulation of "effective connectivity". Journal of neurophysiology. 1989;61(5):900–917. doi:10.1152/JN.1989.61.5.900.

24. Gilbert CD, Wiesel TN. Morphology and intracortical projections of functionally characterised neurones in the cat visual cortex. Nature 1979 280:5718. 1979;280(5718):120–125. doi:10.1038/280120a0.

25. Magrans de Abril I, Yoshimoto J, Doya K. Connectivity inference from neural recording data: Challenges, mathematical bases and research directions. Neural Networks. 2018;102:120–137. doi:10.1016/J.NEUNET.2018.02.016.

26. Denker M, Köhler C, Jurkus R, Kramer M, Kern M, Kurth AC, et al. Elephant 0.12.0. 2023;doi:10.5281/ZENODO.7673930.

27. Chiappalone M, Vato A, Berdondini L, Koudelka-Hep M, Martinoia S. Network dynamics and synchronous activity in cultured cortical neurons. International journal of neural systems. 2011;17(2):87–103. doi:10.1142/S0129065707000968.

28. Spivak L, Levi A, Sloin HE, Someck S, Stark E. Deconvolution improves the detection and quantification of spike transmission gain from spike trains. Communications Biology 2022 5:1. 2022;5(1):1–17. doi:10.1038/s42003-022-03450-5.

29. Stark E, Abeles M. Unbiased estimation of precise temporal correlations between spike trains. Journal of Neuroscience Methods. 2009;179(1):90–100. doi:10.1016/J.JNEUMETH.2008.12.029.

30. English DF, McKenzie S, Evans T, Kim K, Yoon E, Buzsáki G. Pyramidal Cell-Interneuron Circuit Architecture and Dynamics in Hippocampal Networks. Neuron. 2017;96(2):505–520. doi:10.1016/J.NEURON.2017.09.033.

31. Kobayashi R, Kurita S, Kurth A, Kitano K, Mizuseki K, Diesmann M, et al. Reconstructing neuronal circuitry from parallel spike trains. Nature Communications 2019 10:1. 2019;10(1):1–13. doi:10.1038/s41467-019-12225-2.

32. Schreiber T. Measuring Information Transfer. Physical Review Letters. 2000;85(2):461. doi:10.1103/PhysRevLett.85.461.

33. Wollstadt P, Lizier JT, Vicente R, Finn C, Martínez-Zarzuela M, Mediano P, et al. IDTxl: The Information Dynamics Toolkit xl: a Python package for the efficient analysis of multivariate information dynamics in networks. Journal of Open Source Software. 2018;4(34):1081. doi:10.21105/joss.01081.

34. Wibral M, Vicente R, Lindner M. Transfer entropy in neuroscience. Understanding Complex Systems. 2014; p. 3–36. doi:10.1007/978-3-642-54474-3_1/COVER.

35. Shorten DP, Spinney RE, Lizier JT. Estimating Transfer Entropy in Continuous Time Between Neural Spike Trains or Other Event-Based Data. PLOS Computational Biology. 2021;17(4):e1008054. doi:10.1371/journal.pcbi.1008054.

36. Cutts CS, Eglen SJ. Detecting pairwise correlations in spike trains: an objective comparison of methods and application to the study of retinal waves. The Journal of neuroscience : the official journal of the Society for Neuroscience. 2014;34(43):14288–303. doi:10.1523/JNEUROSCI.2767-14.2014.

37. Truccolo W, Eden UT, Fellows MR, Donoghue JP, Brown EN. A point process framework for relating neural spiking activity to spiking history, neural ensemble, and extrinsic covariate effects. Journal of Neurophysiology. 2005;93(2):1074–1089. doi:10.1152/jn.00697.2004.

38. Linderman S, Adams RP, Pillow JW. Bayesian latent structure discovery from multi-neuron recordings. Advances in Neural Information Processing Systems. 2016;29.

39. Apostolopoulou I, Linderman S, Miller K, Dubrawski A. Mutually Regressive Point Processes. Advances in Neural Information Processing Systems. 2019;32.

40. Donner C, Opper M. Inverse Ising problem in continuous time: A latent variable approach. Physical Review E. 2017;96(6):062104. doi:10.1103/PhysRevE.96.062104.

41. Donner C, Opper M. Efficient Bayesian Inference of Sigmoidal Gaussian Cox Processes. Journal of Machine Learning Research. 2018;19:1–34.

42. Daley DJ, Vere-Jones D. An Introduction to the Theory of Point Processes. 2008;doi:10.1007/978-0-387-49835-5.

43. Kobayashi R, Kurita S, Kurth A, Kitano K, Mizuseki K, Diesmann M, et al. Synthetic spike data generated by a network of 1,000 Hodgkin-Huxley type neurons. 2019;doi:10.6084/m9.figshare.9637904.v1.
